# The effect of helminth infection on vaccine responses in humans and animal models: A systematic review and meta‐analysis

**DOI:** 10.1111/pim.12939

**Published:** 2022-07-05

**Authors:** Agnes Natukunda, Ludoviko Zirimenya, Jacent Nassuuna, Gyaviira Nkurunungi, Stephen Cose, Alison M. Elliott, Emily L. Webb

**Affiliations:** ^1^ Immunomodulation and Vaccines Programme MRC/UVRI and LSHTM Uganda Research Unit Entebbe Uganda; ^2^ MRC International Statistics and Epidemiology Group, Department of Infectious Disease Epidemiology London School of Hygiene and Tropical Medicine London UK; ^3^ Department of Infection Biology London School of Hygiene and Tropical Medicine London UK; ^4^ Department of Clinical Research London School of Hygiene and Tropical Medicine London UK

**Keywords:** helminths, immunity, meta‐analysis, systematic review, vaccines

## Abstract

Vaccination has potential to eliminate infectious diseases. However, parasitic infections such as helminths may hinder vaccines from providing optimal protection. We reviewed existing literature on the effects of helminth infections and their treatment on vaccine responses in humans and animals. We searched literature until 31 January 2022 in Medline, EMBASE, Global health, Scopus, and Web of science; search terms included WHO licensed vaccines and human helminth types. Standardized mean differences (SMD) in vaccine responses between helminth infected and uninfected or anthelminthic treated and untreated individuals were obtained from each study with suitable data for meta‐analysis, and combined using a random effects model. Analysis was stratified by whether helminth exposure was direct or prenatal and by vaccine type. This study is registered with PROSPERO (CRD42019123074). Of the 4402 articles identified, 37 were included in the review of human studies and 24 for animal experiments. For human studies, regardless of vaccine type, overall SMD for helminth uninfected/treated, compared to infected/untreated, was 0.56 (95% CI 0.04–1.07 and *I* ^2^ = 93.5%) for direct helminth exposure and 0.01 (95% CI −0.04 to 0.07 and *I* ^2^ = 85.9%) for prenatal helminth exposure. Effects of anthelminthic treatment were inconsistent, with no overall benefit shown. Results differed by vaccine type, with responses to live vaccines most affected by helminth exposure. For animal studies, the most affected vaccine was BCG. This result indicates that helminth‐associated impairment of vaccine responses is more severe for direct, than for prenatal, helminth exposure. Further research is needed to ascertain whether deworming of individuals before vaccination may help improve responses.

## 1 INTRODUCTION

Prevention of diseases through vaccination continues to be a major global health focus and the recent SARS‐CoV‐2 pandemic has further brought this to international public attention. The World Health Organization (WHO) estimated that in 2019, global immunization coverage for diseases such as polio, measles and diphtheria‐tetanus‐pertussis had surpassed 70%.^1^ Although global vaccine coverage is on the rise including in low‐ and middle‐income countries, efficacy and immunogenicity of some vaccines varies greatly by population and geographic location, with impaired responses reported in low‐versus high‐income and rural versus urban settings.^2^, ^3^, ^4^ These settings are characterized by high exposure to infections, including parasites such as helminths and malaria.^5^, ^6^ Exposure to parasitic infections has been proposed to play a role in modulating vaccine immune responses.^7^

Immunomodulation by helminths has been tested for several vaccines in both humans^8^, ^9^ and animal models.^10^, ^11^, ^12^ Many of these studies have reported that vaccine‐specific immune responses may be impaired due to the presence of these infections prior to vaccination. However, some studies have reported improved tetanus, HPV and polio immune responses in individuals exposed to helminths or malaria infections,^13^, ^14^, ^15^ indicating that the effect of these infections on immune responses may vary by vaccine type and individual. Therefore, understanding the effect of helminths on how humans and animals respond to vaccines is an important topic that may have global health policy implications.

The effect of helminth infections on immunization responses has been previously reviewed.^16^, ^17^ The most recent review combined data from both human studies and animal experiments for all vaccine types and concluded that immune responses to vaccines were negatively affected by presence of ‘parasitic’ infections, defined to include helminths, protozoa, bacteria and viruses. Interpretation of these combined results is challenging, since humans may respond differently to parasite exposure than animals, and animal experiments are a more controlled environment. Furthermore, effects may differ depending on the vaccine type. The review highlighted the significance of chronic, rather than acute helminth infections, and evidence of a greater effect on T‐cell dependent vaccines. Elsewhere it is proposed that parasitic infections may be more likely to affect responses to live, than inert, vaccines^4^; responses to orally administered vaccines may also be easily modulated compared to parenterally administered vaccines.^18^ Why some vaccine responses are more affected than others is not fully known, however, helminths may, for example, trigger innate immune response profiles that change how the immune system responds to live vaccines.^19^

The previous review did not examine the effect of prenatal exposure to ‘parasitic’ infections on vaccine responses.^16^ We cannot fully assess the effects of exposure to helminths without assessing effects of prenatal helminth exposure since a significant number of vaccinations happen between birth and 1 year of age when children are less likely to be individually exposed to helminths. Therefore, reviewing existing evidence on the effect of prenatal exposure to helminths on vaccine responses is important.

The objective of this work was to search, review and summarize existing literature on the effect of helminth infections and/or their treatment on vaccine responses in human and animal models separately, assess whether the direction of modulation is vaccine‐specific and assess the effects of both direct helminth exposure and in utero helminth exposure. The purpose of this work was to inform public health policy and identify potential interventions that could improve vaccine effectiveness.

## 2 MATERIALS AND METHODS

### 2.1 Search strategy and selection criteria

The review and meta‐analysis were conducted and reported according to PRISMA guidelines.^20^ Literature searches were conducted up to 31 January 2022 in Medline, EMBASE, Global health, Scopus and Web of Science with no start date limit. The search terms targeted articles reporting the effect of helminths or their treatment on vaccine responses and included all human helminth species and WHO‐licensed vaccines (Appendix 1 in Supporting Information). All retrieved articles from the database searches were exported to Mendeley software for further management. We further searched bibliographies to identify articles that were not captured during the database search.

In the first stage, titles and abstracts of retrieved articles were screened for potential inclusion by two reviewers (AN and LZ) for human studies and two reviewers (GN and JN) for animal studies. The second stage involved reviewing full texts of articles deemed relevant in stage one with the same pairs of reviewers. In both stages, articles were independently reviewed for inclusion by each of the two reviewers; in case of disagreement a third reviewer (EW) was involved to discuss discrepancies and reach consensus. Studies were included in the qualitative and quantitative review if they compared immune responses to a vaccine between helminth infected and uninfected groups or between anthelminthic treated and untreated groups; and if helminth status of study participants was laboratory diagnosed before vaccination and an immunological outcome measured thereafter. Studies were included in the quantitative synthesis if data suitable for a meta‐analysis were reported in the article or made available upon contacting the author. Articles were excluded if the status of helminth infection was not determined or was determined after vaccination had occurred, or if there was no comparative control group or if they described case series. The review included both intervention and observational studies. The review protocol is registered at www.crd.york.ac.uk/prospero, CRD42019123074.^21^

### 2.2 Data analysis

Data from relevant articles were extracted from text, tables and figures (using web plot digitizer version 4.4^22^) into a Microsoft Excel data extraction tool we designed specifically for this purpose. For articles where data extraction failed, authors were contacted to provide the relevant data. Data extracted included study and participant characteristics, vaccines and helminth species, and immunological outcomes. Duplicate articles missed during the automated deduplication process in Mendeley software were identified and excluded at this stage.

We used the Effective Public Health Practice Project tool (EPHPP)^23^ to assess quality of individual human studies. With this tool, studies were rated as strong, moderate or weak based on an eight‐component checklist. The SYRCLE^24^ risk of bias tool was used for animal experiments where studies were rated as having low, high or unclear risk of bias on 10 components. Details of the items scored are in Appendices 2 and 3 in Supporting Information.

The primary outcome for our review was immune response to vaccines. For relevant articles, we extracted a narrative summary of main findings which included all immune parameters reported on in the articles. For the purpose of quantitative synthesis, mean (SD), median (IQR) and geometric mean (95% CI) were extracted separately by helminth infection or treatment status. Summary measures other than mean (SD) were converted to mean (SD) on the log_10_ scale.^25^, ^26^ Where studies reported on multiple immune parameters for the same vaccine, we chose the parameter that is thought to be the best correlate of protection for that vaccine. When the outcome of interest was reported at multiple time points, a weighted average across time points was obtained as a single measure for that study.^27^

Since studies reported several vaccine‐specific immune responses and on different units and scales, standardized mean differences (SMD) between helminth infection and/or treatment groups with 95% confidence intervals were calculated for each study using Hedges' g.^28^ We hypothesised that in addition to the sampling variability that exists within studies, the effect of helminths on vaccine responses would be likely to vary from study to study, therefore, study specific SMDs were averaged into an overall effect size and 95% confidence interval using a random effects model with restricted maximum likelihood estimation, to account for between study variability. The helminth infected/anthelminthic untreated group was used as the reference category, therefore an SMD of >0 represents higher response in the uninfected versus infected, or in the treated versus untreated group. *I* ^*2*^ statistic was used to quantify the amount of heterogeneity among study‐specific SMDs. It ranges between 0% to 100%, with 0% indicating no heterogeneity between study specific SMDs.^29^ Analysis was conducted separately by whether the study reported the effect of direct or prenatal exposure to helminth infection. For direct helminth exposure, individuals are considered to be directly infected with helminths; prenatal exposure is where the subject is exposed to helminths in utero. Subgroup analyses by vaccine type (separately for direct versus prenatal helminth exposure) were conducted to estimate vaccine‐specific SMDs. As a secondary analysis, we present data comparing vaccine responses among helminth uninfected versus infected, and anthelminthic treated versus untreated individuals to evaluate whether the direction of effects of being helminth uninfected and receiving anthelminthic treatment were consistent. Further, sensitivity analysis was done by excluding studies that had extremely small or large effect sizes and/or very small sample sizes. Publication bias was assessed using funnel plots with Egger's test being used to test for funnel plot symmetry. Analysis was done using Stata meta‐analysis suite ‘meta’ in Stata version 16.

## 3 RESULTS

### 3.1 Human studies

**FIGURE 1:**
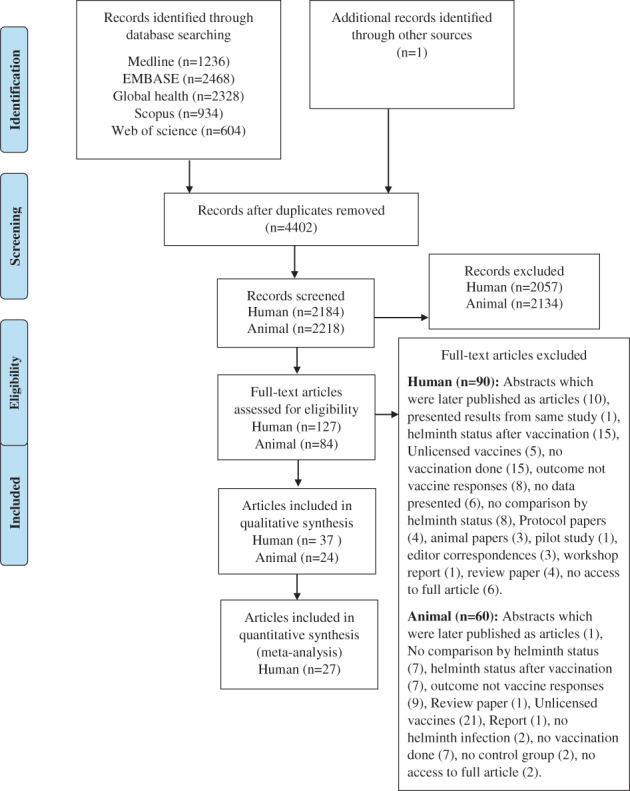
Flow diagram of study selection and screening

Article eligibility screening results and reasons for exclusion are presented in Figure 1. The search identified 2184 unique articles. Of these, 37 (19 from randomized controlled trials) were included in the qualitative review of human studies. Data suitable for meta‐analysis for human studies was available for 27 articles reporting data from 23 studies. Of these, 13 articles evaluated the effect of direct helminth exposure/treatment on vaccine responses and 14 the effect of prenatal helminth exposure/treatment on vaccine responses. Articles included in the human review and meta‐analysis were from research conducted in Africa, Asia and South America and were published between 1983 and 2021. Relevant articles reported data on a total of 14 vaccines with many articles reporting data on multiple vaccines: BCG (11 articles), tetanus toxoid (14), diphtheria (6), influenza (7), hepatitis B (7), pertussis (2), measles (6), polio (3), meningococcal (1), pneumococcal (2), oral typhoid (2), cholera (1), rubella (1) and rotavirus (1). Since we hypothesised different effects on different vaccine types, results are presented separately for each vaccine. For each of the vaccines included in the meta‐analysis, details of study‐specific SMDs, their contribution to the overall SMD and heterogeneity measure (*I* ^*2*^) are presented in Appendix 4 in Supporting Information for direct helminth exposure and Appendix 5 in Supporting Information for prenatal helminth exposure. A narrative summary of findings including study characteristics and references from human studies is presented in Appendix 6 in Supporting Information.

**FIGURE 2:**
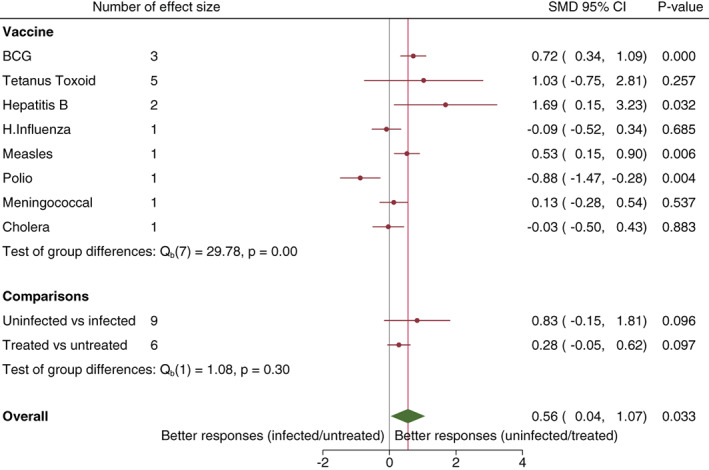
Forest plot of the effect of direct helminth infection or anthelminthic treatment on vaccine responses. References and study specific standardized mean differences are presented in Appendix 4 in Supporting Information.

**FIGURE 3:**
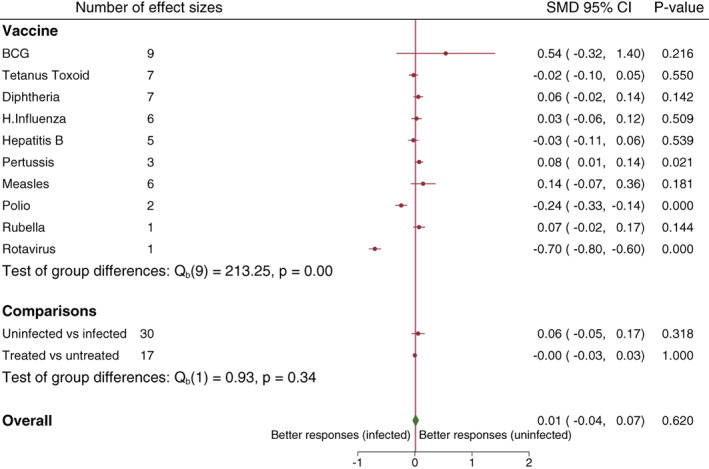
Forest plot of the effect of prenatal helminth infection or anthelminthic treatment on vaccine responses. References and study specific standardized mean differences are presented in Appendix 5 in Supporting Information.

Of 11 articles that reported on BCG, four reported on the effect of direct helminth exposure on vaccine responses and three of these were eligible for meta‐analysis. The meta‐analysis results show evidence of higher immune responses to BCG among helminth uninfected compared to infected individuals (SMD 0.72, 95% CI 0.34 to 1.09) (Figure 2). The fourth article whose data was not suitable for meta‐analysis also reported higher responses in persons uninfected with *Onchocerca volvulus* ^30^ (Appendix 6 in Supporting Information). Seven articles contributing nine effect sizes were included in both the narrative summary and the meta‐analysis for prenatal helminth exposure and the average effect size was SMD 0.54, 95% CI −0.32 to 1.40 (Figure 3). Since this analysis included two articles from the same study that reported the same outcomes at 1 year^31^ and 5 years,^32^ a sensitivity analysis excluding results from the year five outcomes article was done and resulted in an average estimate of SMD 0.73, 95% CI −0.42 to 1.88. Heterogeneity was moderate for direct helminth exposure (*I* ^2^ = 31%) (Appendix 4 in Supporting Information) and large for prenatal exposure (*I* ^2^ = 99%) (Appendix 5 in Supporting Information).

Of 14 articles that reported on tetanus toxoid, seven reported on the effect of direct helminth exposure on vaccine responses and five of these were eligible for meta‐analysis. Meta‐analysis results show no overall significant helminth effect on TT responses (SMD 1.03, 95% CI −0.75 to 2.81 and *I* ^2^ = 98%) (Figure 2). Two articles not included in the meta‐analysis reported significant higher antibody responses in helminth uninfected compared to infected individuals.^33^, ^34^ The average effect size for association between prenatal helminth exposure and TT responses among six articles contributing seven effect sizes was (SMD −0.02, 95% CI −0.10 to 0.05 and *I* ^2^ = 39%) (Figure 3). One article not included in the meta‐analysis reported no effect of prenatal anthelminthic treatment on TT responses.

All six relevant articles on diphtheria contributing seven effect sizes were included in the meta‐analysis and all were investigating the effect of prenatal helminth exposure. Of these, five effect sizes came from studies that looked at the effect of prenatal helminth exposure and two effect sizes were from a study that evaluated effect of prenatal treatment of helminths on vaccine responses. The two effect sizes from this study resulted from two independent randomisations of treatment with albendazole versus placebo and treatment with praziquantel versus placebo.^31^ Overall, the average effect was (SMD 0.06, 95% CI −0.02 to 0.14 and *I* ^2^ = 43%) (Figure 3).

Among seven articles that reported on influenza vaccine, one article reported on direct helminth exposure and found that treatment of helminths before vaccination was not significantly associated with vaccine specific immune responses to influenza vaccine.^35^ The average effect for the five articles reporting on prenatal helminth exposure was SMD 0.03, 95% CI −0.06 to 0.12 and *I* ^2^ = 52% (Figure 3). The sixth article not included in the meta‐analysis reported higher titers at 12 months among children of uninfected mothers compared to infected untreated and infected treated groups; no significant differences were observed at 6 months.^36^

In seven studies on Hepatitis B, meta‐analysis of results averaged from two studies reported higher responses among helminth uninfected individuals (SMD 1.69, 95% CI 0.15 to 3.23 and *I* ^2^ = 94%) (Figure 2). One study not included in the meta‐analysis found no significant difference in anti‐hepatitis B titers between helminth infected and uninfected individuals.^37^ Among the four studies investigating prenatal exposure to helminths, there was no overall association with Hepatitis B responses (SMD −0.03, 95% CI −0.11 to 0.06 and *I* ^2^ = 21%) (Figure 3).

Among six articles that reported on measles, we identified only one article on direct helminth exposure which reported significantly higher responses among helminth uninfected compared to infected individuals 1 week after immunization and no significant difference at 24 weeks post immunization.^38^ Six effect sizes from five articles were included in the meta‐analysis for the effect of helminth infection on responses to measles among children exposed to helminths prenatally. The average effect size was (SMD 0.14, 95% CI −0.07 to 0.36 and *I* ^2^ = 92%) (Figure 3). One of these studies contributed two effect sizes to the meta‐analysis; one effect size for treatment with albendazole versus placebo and the other for treatment with praziquantel versus placebo.^31^

The effect of prenatal helminth exposure on responses to pertussis vaccine was reported in two articles. The average effect size was (SMD 0.08, 95% CI 0.01 to 0.14 and *I* ^2^ = 14%). One article each identified for rubella,^15^ meningococcal^39^ and cholera^39^ vaccines showed no association with helminth infection status. We found one article on the effect of direct helminth exposure on live, oral polio vaccine which reported lower responses among uninfected compared to infected individuals.^40^ Similarly, findings from two articles reporting on the effect of prenatal helminth exposure on live, oral polio vaccine suggested lower responses among children of mothers without helminths (SMD −0.24, 95% CI −0.33 to −0.14 and *I* ^2^ = 0.01%). The only study we found on live, oral rotavirus also reported significantly lower response levels in children born to helminth uninfected compared to infected mothers.^15^

As an exploratory analysis, we computed overall SMDs separately for articles reporting on the effect of helminth infection and articles reporting on the effect of anthelminthic treatment. We found that there was no significant difference in vaccine responses between helminth infected and uninfected (SMD 0.83, 95% CI −0.15 to 1.81) or anthelminthic treated and untreated (SMD 0.28, 95% CI −0.05 to 0.62). When the overall SMD resulting from articles reporting on the effect of helminth infection was compared to the overall SMD from articles reporting on the effect of anthelminthic treatment, there was little statistical evidence for a difference between the two overall SMDs (*p* = .30) (Figure 2). For prenatally helminth exposed children, there was no significant difference in vaccine responses between helminth infected and uninfected (SMD 0.06, 95% CI −0.05 to 0.17) or anthelminthic treated and untreated (SMD 0.00, 95% CI −0.03 to 0.03) groups and there was no statistical evidence for a difference between the two overall SMDs (*p* = .34) (Figure 3). Overall, combining results from all vaccines, we found significantly higher vaccine responses among direct helminth uninfected/treated compared to helminth infected/untreated individuals (SMD 0.56, 95% CI 0.04 to 1.07 and *I* ^2^ = 93.5%) (Appendix 4 in Supporting Information) and no significant association of prenatal helminth infection/treatment with vaccine responses (SMD 0.01, 95% CI −0.04 to 0.07 and *I* ^2^ = 85.9%) (Appendix 5 in Supporting Information).

### 3.2 Animal studies

The database search identified 2218 unique animal experiment articles and of these 24 articles were included in this review (Figure 1). Identified relevant articles assessed the effect of helminths on BCG (11 articles), tetanus toxoid (2), diphtheria (1), influenza (2), hepatitis B (2), pertussis (1), pneumococcal (1), HPV (1), yellow fever (1), cholera (1) and rabies (1). Reviewed articles were published between 1969 and 2021. For animal experiments, a meta‐analysis was not done due to few studies per vaccine type. A narrative summary of results including study characteristics and references of articles for animal experiments is presented in Appendix 7 in Supporting Information.

Of the 11 articles that presented data on BCG, 10 reported a reduction in some form of BCG response among helminth infected compared to uninfected animals. Responses reported in these studies included antibody and cytokines,^10^, ^41^, ^42^, ^43^, ^44^ mycobacterial clearance in the lungs,^45^ lymph node expansion,^46^ hypersensitive footpad swelling,^47^, ^48^ intestinal secretion and absorption and survival time of animals.^49^ One experiment in wild mice found no effect of chronic helminth infection on either primary or memory T regulatory cell response, progression to *Mycobacterium tuberculosis* infection and BCG efficacy.^50^

Data on other vaccines showed harmonious results with evidence of impaired antibody or cytokine responses among helminth infected compared to uninfected animals for tetanus,^51^, ^52^ diphtheria,^53^ influenza,^8^, ^54^ hepatitis B,^11^, ^55^ pertussis,^56^ pneumococcal,^57^ HPV,^12^ yellow fever,^58^ cholera^59^ and rabies^60^ vaccines. The stage of parasite infection seemed to play a role in whether a difference was found, for instance in two studies on hepatitis B and tetanus, there was no significant difference in vaccine responses between the groups when *Trichinella spiralis* infection was in muscle stage^55^ or when vaccination was done in the prepatent period (1–6 weeks after *Schistosoma mansoni* infection).^52^

### 3.3 Quality assessment

Risk of bias assessment for human studies showed there was significant underreporting or lack of blinding of outcome assessors. Among the included articles, only 16% reported blinding for both participants and outcome assessors. Taking into consideration all eight risk of bias components, 18 (49%) articles had a moderate or strong rating (Appendix 2 in Supporting Information). For animal studies, studies frequently did not report whether there was allocation concealment, blinding of outcome assessors, random allocation of animals to intervention arms, or whether animals were housed randomly during the experiment. For each of these components, more than 80% of studies had a high or unclear risk of bias (Appendix 3 in Supporting Information). Funnel plots and Egger's test indicated the presence of publication bias (Appendix 8 in Supporting Information for direct helminth exposure studies and Appendix 9 in Supporting Information for prenatal helminth exposure studies). A sensitivity analysis excluding one extremely large effect size each from direct helminth exposure^61^ and from prenatal helminth exposure risk of bias analyses^62^ changed Egger's test *p* values from .001 to .523 for direct helminth exposure and from <.001 to .101 for prenatal helminth exposure.

## 4 DISCUSSION

We have presented results of a narrative summary and meta‐analysis on the effect of helminths on vaccine responses for human studies and a narrative summary of findings for animal studies. Results from the meta‐analysis show that, when data on all vaccines were combined, established helminth infection at the time of vaccination affects vaccine‐specific immune responses. These findings are consistent with another review that investigated the effect of ‘parasitic’ infections on vaccines.^16^ However, the patterns and mechanisms involved are complex and differ depending on the type of vaccine, the helminth species and whether it is direct helminth infection/treatment or prenatal infection/treatment that is being assessed. The results show that direct helminth exposure reduced responses to BCG and measles vaccines, both of which are live vaccines, although only one article was identified for measles. It has been shown that live vaccines may be more likely to be negatively affected by presence of helminths^4^ and this may explain the results we observed. Hepatitis B, a non‐live vaccine was also negatively affected as shown from two studies. When data from all vaccines were combined, we did not find evidence that prenatal helminth exposure/treatment significantly affected responses to vaccines overall, although meta‐analysis results for pertussis showed an adverse association with maternal helminth infection, whilst a meta‐analysis for live, oral polio vaccine (and one study on live, oral rotavirus vaccine) showed higher vaccine responses among infants of infected mothers. Because of a small number of articles per vaccine, vaccine specific results should be interpreted with caution.

Results from animal studies showed that helminth infection at the time of vaccination reduced responses to BCG. Although we found few studies for other vaccines (tetanus, diphtheria, influenza, hepatitis B, pertussis, pneumococcal, HPV, yellow fever and cholera), results from these studies reported impaired vaccine specific responses due to helminth infection. Results from animal experiments were more consistent than for humans. A possible explanation for this is that in mice, the helminth infection is controlled in terms of dose and timing, intensity of infection may be greater than in otherwise healthy human subjects, and (except in wild mice) issues of confounding with other environmental exposures and factors such as nutrition are avoided.

The negative effect of helminths on BCG responses was consistent for studies of direct helminth exposure. The findings observed from human studies are supported by earlier experiments in mice that showed reduced purified protein derivative (PPD)‐specific in‐vitro interferon gamma,^41^, ^42^, ^43^ lymph node expansion^46^ and delayed hypersensitivity in footpad swelling^47^ among helminth‐infected mice. In one experiment where mice were challenged with tubercle bacilli after immunization, helminth‐infected mice died earlier than uninfected mice.^49^ Studies that investigated the effect of prenatal helminth exposure generally found no associations with BCG‐specific immune responses. These findings emphasize the importance of giving BCG at birth and have implications for the use of BCG ‘booster’ vaccination.

When we looked at studies investigating the effect of direct helminth exposure on Hepatitis B responses, we found that infection before vaccination significantly impaired responses to Hepatitis B. This was based on data from only two studies and there was substantial heterogeneity among SMDs, although all estimates showed the same direction of effect. We did not find evidence of an effect of prenatal helminth exposure on responses to Hepatitis B vaccine.

In our analysis, most vaccines were not significantly affected by prenatal helminth infection/treatment, however we found that for pertussis there was some evidence from meta‐analysis of two studies that vaccine responses were reduced among infants of infected/untreated mothers. On the other hand, based on results from two studies, vaccine specific responses to live, oral polio were higher among infants of helminth infected/untreated compared to uninfected/treated mothers and, interestingly, the one study on live, oral rotavirus vaccine in infants showed a similar effect.

The findings from studies on tetanus toxoid and direct helminth exposure showed conflicting results, however the results indicated that on average TT vaccine responses were not significantly affected by helminth infection. A sensitivity analysis excluding one study with very small sample size and a large effect size did not alter this conclusion. However, an animal experiment involving a Swiss mouse found that prolonged infection with *Schistosoma mansoni* before vaccination resulted in lower antitoxin titres.^52^ This accords with evidence that chronic helminth infection alters vaccine responses more than acute helminth infection.^16^ Similarly, for human studies, overall we found no evidence that prenatal exposure to helminths affected responses to TT.

For many vaccines included in the human study review, that is, measles, influenza, meningococcal, oral typhoid, polio and cholera we found either only one article or could not obtain data suitable for a meta‐analysis. This emphasizes that there are research gaps relating to many common vaccines. The findings from the individual studies reported differing results with some studies reporting significant reduction in responses due to helminth infection^35^, ^63^ and others no effect.^39^, ^64^ Results from single studies should be interpreted with caution as they may not be generalizable.

In our meta‐analysis, we found large heterogeneity between studies for some vaccine types and we could not interrogate this further using meta regression due to the small number of studies. However, heterogeneity was low to moderate for BCG for direct helminth exposure studies and for tetanus, diphtheria, Hepatitis B, pertussis and polio for prenatal helminths exposure studies. Despite large heterogeneity between studies for some vaccines, these findings are still relevant as they show the average effect of helminths on vaccine responses and also highlight the diverse situations in which studies on this topic are designed and conducted. Differences in geographical locations, varying follow‐up periods, timing of measurement of responses after vaccination, choice of outcome measure to assess, length of period between anthelminthic treatment and vaccination, method of helminth diagnosis, type of helminth and location of helminth in the body (i.e., blood, tissue, or gut), all of which vary from study to study, may all contribute to explain this variability. In our analysis, we acknowledge the presence of the unexplained heterogeneity between the studies and used a random effects model that takes this into account whilst estimating the average effect. Also, in the meta‐analysis, we included responses to vaccines that are thought to be the best correlates of protection. However, we have noted several studies where responses other than antibody responses were significantly affected by presence of helminth infection. This may have an impact on the overall interpretation of our results. Also, for some helminth ‘mass treatment’ studies (where participants were randomized regardless of baseline infection status), the prevalence of helminths at baseline was low which potentially underestimated the effect of anthelminthic treatment on vaccine responses. Furthermore, different helminths may affect vaccine responses differently; however, it was not possible to investigate this since most studies reported infection with multiple helminths. A large percentage of studies included in our review and meta‐analysis did not report on blinding of outcome assessors during the conduct of the studies which left unanswered the question of whether there was no blinding at all or if it was simply not reported. It is possible that articles published in languages other than English could have been missed even when the literature search was not restricted to articles published in English. This review did not look at studies where helminth infection is determined after vaccination and how this might affect already established immune responses, this is a question that remains to be addressed in future reviews. We acknowledge that co‐infections with other parasites may confound the relationship between helminths and vaccine responses, however we did not investigate this further due to limited data reported on such infections. Lastly, we did not include investigational vaccines because assessment of helminth infection is seldom included in trial protocols even in endemic settings, and furthermore, early phase vaccine trials often include a different age group (with different helminth exposure) to the eventual target age group for the vaccine. Results of this review suggest that assessment for helminths should be considered, especially for vaccines that will be used, and often most needed, in helminth‐endemic settings.

## 5 CONCLUSION

Overall, we found that helminths interfere with some vaccine responses, with more consistent results from animal studies than from studies in humans. Further, it is clear that the effect of helminths on some vaccines such as BCG and Tetanus Toxoid has been investigated more than other vaccines. For the less investigated vaccines, little is known about the impact of helminths on response to these vaccines. With this review and meta‐analysis, we have presented evidence that established helminth infection at the time of vaccination impairs responses to BCG and Hepatitis B vaccines in humans and several vaccines are affected in animals. Furthermore, in humans, these effects are predominantly seen among individuals directly exposed to helminths rather than helminth exposure in utero. The findings presented here suggest that treatment of direct helminth infection before vaccination may help improve responses. However, stronger trials are needed to inform government policy regarding the need for treatment of worms before immunization. Consideration of helminths and other co‐infections in early‐phase trials of new vaccines intended for helminth‐endemic settings may be beneficial.

## AUTHOR CONTRIBUTIONS

Alison M. Elliott conceived the idea. Agnes Natukunda conducted the literature searches, Agnes Natukunda, Ludoviko Zirimenya, Gyaviira Nkurunungi, Jacent Nassuuna screened articles for relevance and conducted the subsequent data extraction, Emily L. Webb was the third reviewer in case of disagreement between the two reviewers each for human and animal studies, respectively. Agnes Natukunda conducted data analysis, Agnes Natukunda, Emily L. Webb and Alison M. Elliott contributed to data interpretation, Agnes Natukunda drafted the manuscript. All authors reviewed, provided input and approved the final version of the manuscript.

## FUNDING INFORMATION

Medical Research Council of the United Kingdom, Grant Number: MR/R02118X/1.

## CONFLICT OF INTEREST

The authors declare no conflict of interest.

### PEER REVIEW

The peer review history for this article is available at https://publons.com/publon/10.1111/pim.12939.

## Supporting information


**Appendix S1**. Supporting Information

## Data Availability

Data sharing is not applicable to this article as no new data were created or analyzed in this study.
