# Supplementary material for: The effect of helminth infection on vaccine responses in humans and animal models: A systematic review and meta‐analysis
Source: Parasite Immunol. 2022 Jul 5;44(9):e12939. doi: 10.1111/pim.12939 (PMC9542036; doi:10.1111/pim.12939)
Supplement: Supplementary file 1 — Appendix S1. Supporting Information [file PIM-44-e12939-s001.doc]

**Appendix 1**

**Medline search phrase**

1 exp Vaccines/ 251291

2 (Cholera or dengue or diphtheria or hepatitis A or hepatitis B or HBV or hepatitis E or Haemophilus influenzae or influenza or human papilloma virus or HPV or Japanese encephalitis or measles or meningococcus or meningococcal or mumps or pertussis or pneumococcus or pneumococcal or poliomyelitis or polio or rabies or rotavirus or rubella or tetanus or tick-borne encephalitis or tuberculosis or typhoid or varicella or yellow fever).tw. 712244

3 (BCG or bacille calmette-guerin).tw. 25189

4 exp helminths/ 140651

5 (hookworm* or Ancylostom* or uncinariasis or Necator or whipworm* or Trichuris or roundworm* or Ascaris or Schistosoma or bilharzia or Echinococcus or hydatid or Hymenolepis or Rodentolepis or Fasciola or liver fluke* or filariasis or elephantiasis or Wuchereria or Brugia or Mansonella or Diptalonema streptocerca or Onchocerca or Dracunculiasis or Guinea worm* or helminth* or Trichinella or Trichina or Toxocara or worms or worm or nematode*).tw. 143579

6 Immunogenicity, Vaccine/ 2740

7 (Immunogenicity or antigenicity).tw. 52868

8 ((vaccin* or immun* or antibod* or cytokine) adj3 response*).tw. 359641

9 (produc* adj3 (antibod* or cytokine or response*)).tw. 115366

10 1 or 2 or 3 854106

11 4 or 5 202236

12 6 or 7 or 8 or 9 482891

13 10 and 11 and 12 1236

**EMBASE search phrase**

1 exp vaccine/ 404283

2 (Cholera or dengue or diphtheria or hepatitis A or hepatitis B or HBV or hepatitis E or Haemophilus influenzae or influenza or human papilloma virus or HPV or Japanese encephalitis or measles or meningococcus or meningococcal or mumps or pertussis or pneumococcus or pneumococcal or poliomyelitis or polio or rabies or rotavirus or rubella or tetanus or tick-borne encephalitis or tuberculosis or typhoid or varicella or yellow fever).mp. 1170877

3 (BCG or bacille calmette-guerin).mp. 63144

4 exp helminth/ 188896

5 (hookworm* or Ancylostom* or uncinariasis or Necator or whipworm* or Trichuris or roundworm* or Ascaris or Schistosoma or bilharzia or Echinococcus or hydatid or Hymenolepis or Rodentolepis or Fasciola or liver fluke* or filariasis or elephantiasis or Wuchereria or Brugia or Mansonella or Diptalonema streptocerca or Onchocerca or Dracunculiasis or Guinea worm* or helminth* or Trichinella or Trichina or Toxocara or worms or worm or nematode*).mp. 206218

6 immunogenicity/ or exp vaccine immunogenicity/ 71448

7 (Immunogenicity or antigenicity).mp. 110725

8 ((vaccin* or immun* or antibod* or cytokine) adj3 response*).mp. 610363

9 (produc* adj3 (antibod* or cytokine or response*)).mp. 325753

10 1 or 2 or 3 1365240

11 4 or 5 259054

12 6 or 7 or 8 or 9 908495

13 10 and 11 and 12 2468

**Global health search phrase**

1 exp vaccines/ 102348

2 (Cholera or dengue or diphtheria or hepatitis A or hepatitis B or HBV or hepatitis E or Haemophilus influenzae or influenza or human papilloma virus or HPV or Japanese encephalitis or measles or meningococcus or meningococcal or mumps or pertussis or pneumococcus or pneumococcal or poliomyelitis or polio or rabies or rotavirus or rubella or tetanus or tick-borne encephalitis or tuberculosis or typhoid or varicella or yellow fever).mp. 366745

3 (BCG or bacille calmette-guerin).mp. 10809

4 exp helminths/ 234088

5 (hookworm* or Ancylostom* or uncinariasis or Necator or whipworm* or Trichuris or roundworm* or Ascaris or Schistosoma or bilharzia or Echinococcus or hydatid or Hymenolepis or Rodentolepis or Fasciola or liver fluke* or filariasis or elephantiasis or Wuchereria or Brugia or Mansonella or Diptalonema streptocerca or Onchocerca or Dracunculiasis or Guinea worm* or helminth* or Trichinella or Trichina or Toxocara or worms or worm or nematode*).mp. 256331

6 exp immunogenicity/ 1762

7 (Immunogenicity or antigenicity).mp. 86548

8 ((vaccin* or immun* or antibod* or cytokine) adj3 response*).mp. 128271

9 (produc* adj3 (antibod* or cytokine or response*)).mp. 22030

10 1 or 2 or 3 409571

11 4 or 5 256700

12 6 or 7 or 8 or 9 203329

13 10 and 11 and 12 2328

**Scopus search phrase**

1. (BCG or {bacille calmette-guerin} or Cholera OR dengue or diphtheria or {hepatitis A} or {hepatitis B} or HBV or {hepatitis E} or {Haemophilus influenza} or influenza or {human papilloma virus} or HPV or {Japanese encephalitis} or measles or meningococcus or meningococcal or mumps or pertussis or pneumococcus or pneumococcal or poliomyelitis or polio or rabies or rotavirus or rubella or tetanus or {tick-borne encephalitis} or tuberculosis or typhoid or varicella or {yellow fever})

2. (hookworm* or Ancylostom* or uncinariasis or Necator or whipworm* or Trichuris or roundworm* or Ascaris or Schistosoma or bilharzia or Echinococcus or hydatid or Hymenolepis or Rodentolepis or Fasciola or {liver fluke} or filariasis or elephantiasis or Wuchereria or Brugia or Mansonella or {Diptalonema streptocerca} or Onchocerca or Dracunculiasis or {Guinea worm*} or Trichinella or Trichina or Toxocara or helminth* or worms or worm or nematode)

3. ((vaccin* or immun* or antibod* or cytokine) W/3 (response*)) OR (produc* W/3 (antibod* or cytokine))

4. 1 AND 2 AND 3 AND 4 = (934)

**Web of science search phrase**

1. (BCG or “bacille calmette-guerin” or Cholera OR dengue or diphtheria or “hepatitis A” or “hepatitis B” or HBV or “hepatitis E” or “Haemophilus influenza” or influenza or “human papilloma virus” or HPV or “Japanese encephalitis” or measles or meningococcus or meningococcal or mumps or pertussis or pneumococcus or pneumococcal or poliomyelitis or polio or rabies or rotavirus or rubella or tetanus or “tick-borne encephalitis” or tuberculosis or typhoid or varicella or “yellow fever”)

2. (“soil transmitted helminths” or hookworm* or Ancylostom* or uncinariasis or Necator or whipworm* or Trichuris or roundworm* or Ascaris or Schistosoma or bilharzia or Echinococcus or hydatid or Hymenolepis or Rodentolepis or Fasciola or “liver fluke*” or filariasis or elephantiasis or Wuchereria or Brugia or Mansonella or “Diptalonema streptocerca” or Onchocerca or Dracunculiasis or “Guinea worm*” or Trichinella or Trichina or Toxocara or helminth* or worms or worm or nematode*)

3. ((vaccin* or immun* or antibod*) NEAR/3 (reaction* or response*)) OR (produc* NEAR/3 (antibod* or cytokine*))

4. 1 (Topic) AND 2 (Topic) AND 3 (Topic) = (604)

**Appendix 2: Risk of bias assessment for human studies**

**
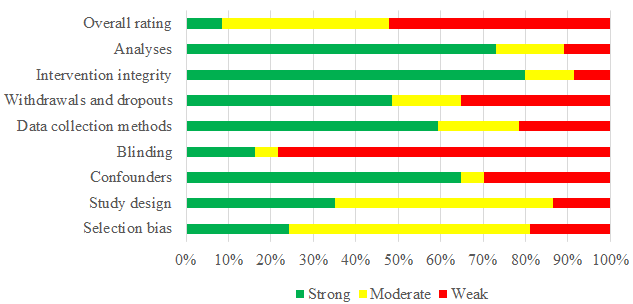
**

The figure shows the percentage of total articles (n=37) included in the human study review categorised as strong, moderate or weak for the 8 components of the EPHPP quality assessment tool. The overall rating for individual studies is in appendix 5 table 1.

**Appendix 3: Risk of bias assessment for animal studies**

**
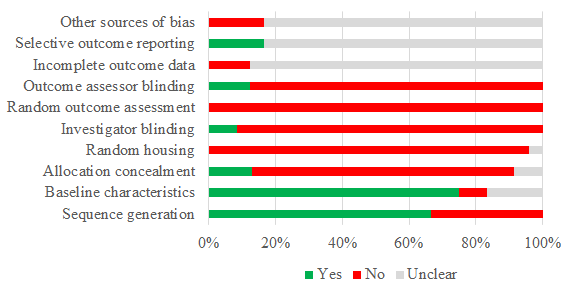
**

The figure shows the percentage of total articles (n=24) included in the animal review categorised as Yes, No or Unclear for the 10 rated items based on the SYRCLE’s risk of bias tool. Where “Yes”, “No” and “Unclear” imply low, high and unclear risk of bias.

**Appendix 4: Forest plot of standardised mean differences (SMD) of direct helminth infection or anthelminthic treatment on vaccine responses.**


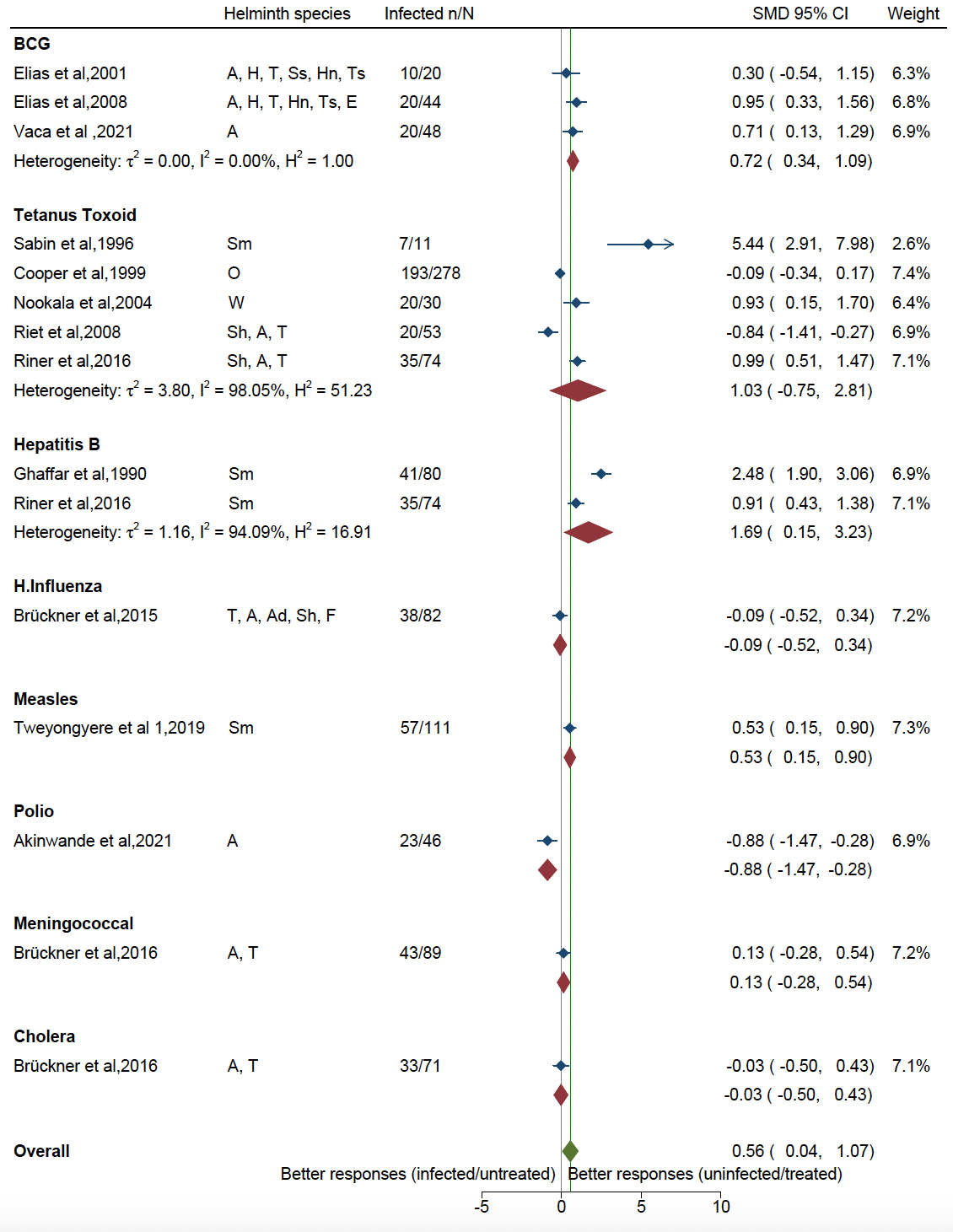


Helminth species A: *Ascaris lumbricoides*, H: Hookworm, T: *Trichuris trichiura*, Ss: *Strongyloides* *stercoralis*, Hn: *Hymenolepis nana*, Ts: *Taenia spp*, E: *Enterobius vermicular*, Sm: *Schistosoma mansoni*, O: *Onchocerca volvulus*, W: *Wuchereria bancrofti*, Sh: *Schistosoma haematobium*, Ad: *Ancylostoma duodenale,* F: Filaria infection

**Appendix 5: Forest plot of standardised mean differences (SMD) of prenatal helminth infection or anthelminthic treatment on vaccine responses.**

**
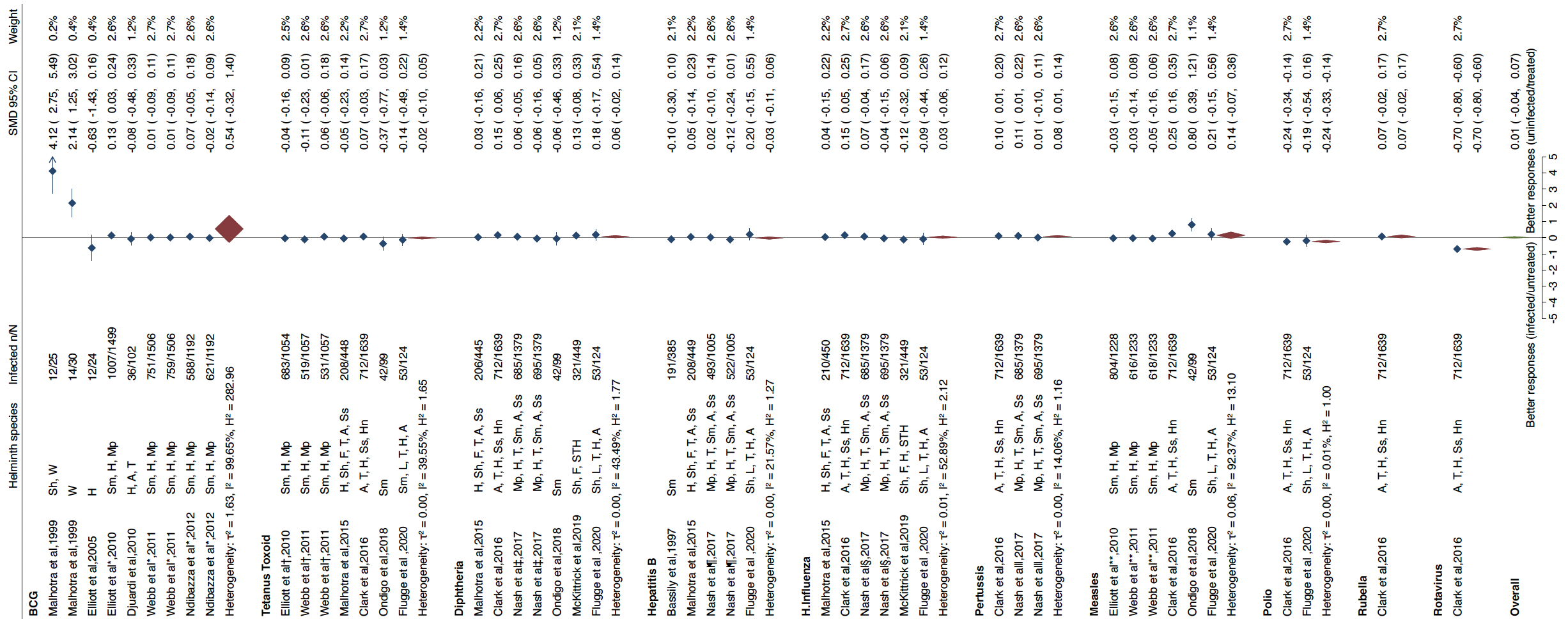
**

* † ‡ § ¶ ** articles from the same study. For articles that appear twice under the same vaccine type, the randomisations were independent for each of the comparisons. Helminth species Sm: *Schistosoma mansoni*, W: *Wuchereria bancrofti*, H: Hookworm, Mp: *Mansonella perstans*, A: *Ascaris lumbricoides*, T: *Trichuris trichiura*, F: *Fasciola hepatica*, Ss: *Strongyloides stercoralis*, Hn: *Hymenolepis* *nana*, L: *Loa loa*, STH: Soil transmitted helminth, Sh: *Schistosoma haematobium*

**Appendix 6: Characteristics and findings from human** studies

| **Author (Year)** | **Study design** | **Population** | **Compared groups (sample size)** | **Age, gender** | **Type of helminth** | **Outcome** | **EPHPP** | **Finding** |
| --- | --- | --- | --- | --- | --- | --- | --- | --- |
| **BCG: Direct helminth exposure** | | | | | | | | |
| *Vaca et al (2012)*[1] | Cohort | Children attending rural schools in the districts of Pedro Vicente Maldonado, Puerto Quito, and San Miguel de Los Bancos in a subtropical region of Pichincha Province | Untreated active infection (20), short term Albendazole treated (13) *vs* long term Albendazole treated (15). 100% *Ascaris lumbricoides* infected at baseline in the untreated and short term treated groups. 6.7% infection in the long term treated group | 8 – 14 year olds, 42% male | *Ascaris lumbricoides* | PPD-specific IFN- ү | M | No effect of short- or long-term treatment of *Ascaris lumbricoide*on on responses to a single booster dose of oral BCG 28 days after vaccination |
| *Elias et al (2008)*[2] | Cohort | Helminth infected Individuals from rural agricultural community of Ginci town, Dendi district, Addis Ababa | Albendazole treated (24) *vs* placebo (20). 61.4% helminth infection at baseline | 13 – 54 year olds, gender not reported | *Trichuris trichiura*, *Ascaris lumbricoides*, Hookworm, *Taenia* spp, *Hymenolepis nana*, *Enterobius vermicularis* | Number of cells secreting the Th1 cytokines IFN-γ and IL-12 | W | Lower number of spot forming units per 2×10^5^ PBMC (SFU) in the placebo group compared to the treated group for both IFN- ү (129 vs 191, p = 0.03 in response to PPD and concanavalin A) and IL-12 in response to PPD; no difference in response to Con A. |
| *Elias et al(2001)*[3] | Randomised placebo-controlled trial | College students from Addis Ababa, Ethiopia | Albendazole treated (10) *vs* Placebo (10). 26.7% helminth infection at baseline | 18 - 24-year-olds, gender not reported | *Ascaris lumbricoides*, Hookworm, *Trichuris trichiura*, *Strongyloides stercoralis*, *Hymenolepis nana*, *Taenia* spp | PPD-specific IFN- ү | W | Higher PPD and PHA-specific IFN- ү in the treated group after BCG compared to placebo (p= 0.02). |
| *Kilian et al(1989)*[4] | Case control | Persons from rural rain forest of Liberia | Helminth infected (21) *vs* uninfected (26) persons | 2 - 55-year-olds, gender not reported | *Onchocerca volvulus* | Tuberculin conversion rate | W | Lower responsiveness in persons infected with *Onchocerca volvulus* compared to uninfected. Conversion rates of 48% vs 85% (p 0.03). |
| **BCG: Prenatal helminth exposure** | | | | | | | | |
| *Badawy et al (2013)*[5] | Cohort | Neonates recruited from a hospital in Mansoura, Egypt | Children of Helminth infected (63) *vs* uninfected (187) mothers | Neonates, 56.4% male | *Schistosoma mansoni*, *Wuchereria bancrofti,* filaria | IgE | W | Elevated IgE levels (p<001) among offspring of helminth infected compared to uninfected mothers. |
| *Ndibazza et al* (2012)*[6] | Randomised placebo-controlled trial | Mother child pairs from Entebbe, Uganda | Children of Albendazole and Praziquantel (500), Praziquantel only (496), Albendazole only (504) *vs* placebo (496) mothers. 68% helminth infection at baseline | 5 year olds, 51.6% male | Hookworm, *Mansonella perstans*, *Schistosoma*  *mansoni* | BCG-specific IFN-γ, IL-5, IL-13, IL-10 and IgG) | S | No effect of maternal treatment during pregnancy on vaccine outcomes at 5 years |
| *Webb et al* (2011)*[7] | Randomised, placebo-controlled trial | Mother child pairs from Entebbe, Uganda | Children of Albendazole and Praziquantel (628), Praziquantel only (626), Albendazole only (625) *vs* placebo (628) mothers. 68% helminth infection at baseline | One year olds, 50% male | Hookworm, *Mansonella perstans*, *Schistosoma*  *mansoni* | IFN- γ, IL-5, IL-13, IL-10 responses to cCFP | S | No difference in cCFP-specific IFN- γ, IL-5, IL-13, IL-10 between infants of albendazole/praziquantel treated and untreated mothers. |
| *Elliott et al* (2010)*[8] | Observational cohort analysis nested within a randomised, placebo-controlled trial | Mother child pairs from Entebbe, Uganda | Children of helminth infected *vs* uninfected mothers. Total sample 1506 | One year olds, 50% male | Hookworm, *Mansonella perstans*, *Schistosoma*  *mansoni* | IFN- ү, IL-5, IL-10, IL-13 responses to cCFP | M | Higher IL-10 responses among offspring of *Mansonella perstans* infected mothers; no significant differences in IFN- γ, IL-5 and IL-13 responses. No effect of other maternal helminth infections. |
| *Djuardi et al (2010)*[9] | Cohort | Mother child pairs from peri-urban Indonesia | Children of helminth infected (38) *vs* uninfected (75) mothers | Neonates, 50% male | Hookworm, *Ascaris lumbricoides*, *Trichuris trichiura* | IL-10, TNF-α, IFN-ү, IL-5, IL-13 responses to PPD | W | No clear effect of maternal intestinal helminth infection status on the response of children to PPD in terms of Th1, Th2, pro or anti-inflammatory cytokines either at pre or at different ages after vaccination |
| *Elliott et al (2005)*[10] | Randomised controlled trial | Mother child pairs from Entebbe, Uganda | Children of Albendazole treated (32) vs placebo (31) mothers. At least 38% helminth prevalence at baseline | One year olds, gender not reported | Hookworm | IFN-γ and IL-5 responses to cCFP | M | Maternal albendazole not significantly associated with IFN-γ (p 0.20) or IL-5 responses. Higher IFN-γ responses to cCFP in infants of hookworm infected mothers (adjusted OR 17.65 (1.20–258.66); p = 0.013). |
| *Malhotra et al (1999)*[11] | Cohort and cross sectional | Mother child pairs from Msambweni Hospital and rural Darigube and Eshu, Kenya | 10 – 14 month olds  Children of helminth infected (12) *vs* uninfected (13) mothers  2-10 year olds  Children of helminth infected (17) *vs* uninfected (16) mothers | 10 - 14 month olds  and 2-10 year olds,  gender not reported | *Wucheria bancrofti*, *Schistosoma haematobium* | IFN- ү and IL-5 responses to PPD | W | PPD-driven T cell IFN- ү production 26-fold higher (p<0.01) at 10–14 months after BCG vaccination for infants who were not sensitized to filariae or schistosomes in utero relative to sensitized infants. Lower IL-5 levels (p 0.02) among non-sensitized children. Higher IFN- ү levels (p 0.008) among 2-10 year olds of uninfected mothers, lower IL-5 (p 0.03) among children of uninfected mothers compared to infected mothers. |
| **Tetanus Toxoid : Direct helminth exposure** | | | | | | | | |
| *Riner et al (2016)*[12] | Cohort | University students potentially from diverse geographical regions of Kenya | Helminth infected (38) *vs* uninfected (99) adults | 18 - 57 year olds, 47% male | *Schistosoma*  *mansoni* | Antibody (IgG) and cytokine (IFN- γ, IL-5) responses to TT | W | Higher IL-5 levels among Sm positive 8 months after boost compared to Sm negative controls (p 0.03). No significant differences observed for IFN- γ and IL-10. Lower TT antibody levels among Sm positive 6 weeks (p<0.002) and 8 months (p 0.07) after boost compared to Sm negative controls in individuals in need of immediate boost. |
| *Riet et al (2008)*[13] | Cohort | School children (7–12 years old) from Nzilé, a rural area and Lambaréné, a semi-urban area of Gabon | Children from rural(all infected with at least one helminth)(20) *vs* semi urban (39.3% infected with any helminth)(33) | 7 - 12 year olds, 41.5% male | *Schistosoma haematobium*, *Ascaris lumbricoides*, *Trichuris trichiura* | Antibody (total IgG and IgG subclasses) and cytokine (IFN- γ, IL-5) responses to TT | W | Higher IgG1(p<0.05), IgG3(p<0.05), IL-5(p<0.01) levels among rural compared to semi urban children. Lower IFN- γ(p<0.05) among rural compared to semi urban children. No difference observed for IgG, IgG2 and IgG4. Semi-urban children with helminth infections were compared to the rural children, a significant difference in anti-IgG3 levels after vaccination was found (p < 0.05). |
| *Nookala et al (2004)*[14] | Cohort | Individuals from Chennai, India | Adults were endemic normal(EN)(10), microfilaremics(CP)(20), symptomatic microfilaremics(MF)(20) | 20 - 66 year olds, 72% male | *Wuchereria bancrofti* | IFN-γ, IL-10 and IgG responses to TT | W | Higher percentage change in IFN-γ responses from baseline among EN (400%) compared to CP (200%, p < 0.05) and MF (200%, p 0.0025). Lower percentage change in IL-10 from baseline value among EN compared to MF (p 0.04). No differences observed for IgG |
| *Cooper et al (1999)*[15] | Cohort | Adults and children from Rio Cayapas in the Santiago River Basin of Esmeraldas  Province, Ecuador | Helminth infected(193) vs uninfected(85) | 5 - 80 year olds, 51.8% male | *Onchocerca volvulus* | Antibody (total IgG, IgG1, IgG2, IgG3, IgG4, IgE) responses to TT | M | Lower mean percentage increase in IgG2 (p <0.05) and IgG3 (p<0.001) among the infected compared to uninfected. No significant differences observed for IgG, IgG1, IgG4 and IgE |
| *Cooper et al (1998)*[16] | Cohort | Adult volunteers from Rio Cayapas in the Santiago River Basin of Esmeraldas Province, Ecuador | Helminth infected(19) *vs* uninfected adults(20) | 15 - 75 year olds, 61.5% male | *Onchocerca volvulus* | IFN-γ, IL-10 and IgG responses to TT | M | Higher percentage change in IFN-γ responses from baseline among uninfected compared to helminth infected (p 0.03). Lower percentage change in IL-10 from baseline value among uninfected (p <0.001). Higher antibody levels in uninfected group (p<0.05) |
| *Sabin et al (1996)*[17] | Cohort | Persons from Caatinga do Moura,  Brazil | Light(2), Moderately Infected(5) *vs* uninfected(3) | 11 - 58 year olds, 31.5% male | *Schistosoma mansoni* | TT-specific IFN- γ | M | Lower IFN- γ among moderately infected (p 0.0031) and lightly infected (p 0.04) persons compared to uninfected persons. |
| *Prost et al (1983)*[18] | Cohort | Residents of Garango district, Burkina Faso | Helminth infected(28) *vs* uninfected(27) persons | 9 - 34 year olds, 41.8% male | Onchocerciasis | Antibody responses to TT | W | Lower antibody responses (p<0.001) among infected compared to uninfected persons. |
| **Tetanus Toxoid : Prenatal helminth exposure** | | | | | | | | |
| *Flügge at al (2020)*[19] | Cohort | Mother child pairs from Lambaréné, Gabon | Children of Helminth infected (53) *vs* uninfected (71) mothers | Neonates, 50.8% male | *Schistosoma haematobium, Loa loa, Trichuris trichiura,* hookworm, *Ascaris lumbricoides* | TT-specific IgG | W | No significant difference in IgG levels between children of helminth infected and uninfected mothers at both 9 and 12 months old |
| *Ondigo et al (2018)*[20] | Cohort | Pregnant women and their children from western Kenya | Children of helminth infected(42) *vs* uninfected(57) mothers | 2 year olds, gender not reported | *Schistosoma mansoni* | TT-specific IgG | W | No significant differences in anti-TT IgG between children of infected and uninfected mothers. |
| *Clark et al (2016)*[21] | Cohort | Mothers and their newborns recruited from Hospital Padre Alberto Buffoni (HPAB) in Quinindé,  Esmeraldas Province, Ecuador | Children of helminth infected(712) *vs* uninfected(927) mothers | 13 months old, 50.5% male | *Ascaris lumbricoides*, *Trichuris trichiura*, Hookworm, *Strongyloides stercoralis*, *Hymenolepis nana* | TT-specific IgG | M | No significant difference (p 0.5792 ) in TT specific IgG levels between the two groups |
| *Malhotra et al (2015)*[22] | Cohort | Healthy pregnant women and their offspring born at the Msambweni District Hospital on the south coast of Kenya | Children of helminth infected *vs* uninfected mothers. Total sample 450 | 6 - 36 months, gender not reported | Lymphatic filariasis, *Schistosoma haematobium*, Hookworm, *Trichuris trichiura, Ascaris lumbricoides, Strongyloides stercoralis* | TT-specific IgG | W | No significant differences in antibody responses between offspring of helminth infected and uninfected groups |
| *Ndibazza et al*^†^ *(2012)*[6] | Randomised Controlled Trial | Mother child pairs from Entebbe, Uganda | Children of Albendazole and Praziquantel (500), Praziquantel only (496), Albendazole only (504) *vs* placebo (496) mothers. 68% helminth infection at baseline | 5 year olds, 51.6% male | Hookworm, *Mansonella perstans*, *Schistosoma*  *mansoni* | TT-specific IFN-γ, IL-5, IL-10, IL-13 and IgG | S | No effect of maternal treatment during pregnancy on vaccine outcomes at 5 years |
| *Webb et al*^†^ *(2011)*[7] | Randomised, placebo-controlled trial | Mother child pairs from Entebbe, Uganda | Children of Albendazole and Praziquantel (628), Praziquantel only (626), Albendazole only (625) *vs* placebo (628) mothers. 68% helminth infection at baseline | One year olds, 50% male | Hookworm, *Mansonella perstans*, *Schistosoma*  *mansoni* | TT-specific IFN- γ, IL-5, IL-10, IL-13; total IgG, IgG4, IgE | S | Lower IL-5 (p 0·02) and IL-13 (p 0.0005) among infants of albendazole treated hookworm infected mothers compared to placebo. No differences observed for IFN-γ, IL-10, Total IgG, IgG4 and IgE. No effect of maternal treatment among infants of *Schistosoma*  *mansoni* infected mothers. |
| *Elliott et al*^†^ *(2010)*[8] | Observation cohort analysis nested within a Randomised, placebo-controlled trial | Mother child pairs from Entebbe, Uganda | Children of helminth infected *vs* uninfected mothers. Total sample 1506 | One year olds, 50% male | Hookworm, *Mansonella perstans*, *Schistosoma*  *mansoni* | TT-specific IFN- γ, IL-5, IL-10 and IL-13 | M | Higher IL-10 responses among offspring of *Mansonella perstans* infected mothers. No significant differences in IFN- γ, IL-5 or IL-13 responses; other maternal helminth infections showed little effect. |
| **Diphtheria : Prenatal helminth exposure** | | | | | | | | |
| *Flügge at al (2020)*[19] | Cohort | Mother child pairs from Lambaréné, Gabon | Children of Helminth infected (53) *vs* uninfected (71) mothers | Neonates, 50.8% male | *Schistosoma haematobium, Loa loa, Trichuris trichiura,* hookworm, *Ascaris lumbricoides* | DT-specific IgG | W | No significant difference in IgG levels between children of helminth infected and uninfected mothers at both 9 and 12 months old |
| *McKittrick et al (2019)*[23] | Cohort | Pregnant women and their newborn infants enrolled at the Msambweni County  Referral Hospital antenatal clinic in Msambweni, Kenya | Children of prenatal helminth infected(473) *vs* uninfected(36) mothers | 0 - 36 months, 46.4% male | *Schistosoma haematobium*, filarial, hookworm, soil-transmitted helminths | DT-specific IgG | M | Higher IgG levels (p 0.001) from six to twelve months of age among children born to mothers with infection at delivery |
| *Ondigo et al (2018)*[20] | Cohort | Pregnant women and their children from western Kenya | Children of helminth infected(42) *vs* uninfected(57) mothers | 2 year olds, gender not reported | *Schistosoma mansoni* | DT-specific IgG | W | No significant differences in anti-Diphtheria IgG between children of gestation infected and uninfected mothers. |
| *Nash et al (2017)*[24] | Randomised placebo controlled trial | Mother child pairs from Entebbe, Uganda | Children of Albendazole+Praziquantel (348), PZQ only (336), albendazole only (346) *vs* placebo (349) mothers. 68% helminth infection at baseline | One year olds, gender not reported | *Mansonella perstans*, Hookworm, *Trichuris trichuria*, *Schistosoma mansoni*, *Ascaris lumbricoides*, *Strongyloides stercoralis* | DT-specific IgG | M | Lower DT responses (p 0.01) among children of Albendazole treated Hookworm infected mothers compared to Albendazole treated hookworm uninfected mothers. |
| *Clark et al (2016)*[21] | Cohort | Mothers and their newborns recruited from Hospital Padre Alberto Buffoni (HPAB) in Quinindé,  Esmeraldas Province, Ecuador | Children of helminth infected(712) *vs* uninfected(927) mothers | 13 month olds, 50.5% male | *Ascaris lumbricoides*, *Trichuris trichiura*, Hookworm, *Strongyloides stercoralis*, *Hymenolepis nana* | DT-specific IgG | M | No significant difference (p 0.2679 ) in DT specific IgG levels between the two groups |
| *Malhotra et al (2015)*[22] | Cohort | Healthy pregnant women and their offspring born at the Msambweni District Hospital on the south coast of Kenya | Children of helminth infected *vs* uninfected mothers. Total sample 450 | 6 - 36 months, gender not reported | Lymphatic filariasis, *Schistosoma haematobium*, Hookworm, *Trichuris trichiura, Ascaris lumbricoides, Strongyloides stercoralis* | DT-specific IgG | W | No significant differences in antibody responses to DT between the groups |
| ***Haemophilus influenza* : Direct helminth exposure** | | | | | | | | |
| *Brückner et al (2015)*[25] | Randomised placebo controlled trial | School children from Lambaréné, Gabone | Anthelminthic treated (50) *vs* placebo (48). 21% helminth infection at baseline | 6 - 10 year olds, 55% male | *Trichuris trichiura*, *Ascaris lumbricoides*, *Ancylostoma duodenale*, *Schistosoma haematobium*, *Fasciola hepatica* | Functional antibody (total IgG1-IgG4, IgA) responses | M | No significant difference observed for vaccine specific IgA and Antigen-specific memory B-cells between the groups. Higher IgG1 (p 0.042) and IgG3(p 0.03) among anthelmintic treated group compared to placebo. |
| ***Haemophilus influenza*: Prenatal helminth exposure** | | | | | | | | |
| *Flügge at al (2020*)[19] | Cohort | Mother child pairs from Lambaréné, Gabon | Children of Helminth infected (53) *vs* uninfected (71) mothers | Neonates, 50.8% male | *Schistosoma haematobium, Loa loa, Trichuris trichiura,* hookworm, *Ascaris lumbricoides* | Hib-specific IgG | W | No significant difference in IgG levels between children of helminth infected and uninfected mothers at both 9 and 12 months old |
| *McKittrick et al (2019)*[23] | Cohort | Pregnant women and their newborn infants enrolled at the Msambweni County  Referral Hospital antenatal clinic in Msambweni, Kenya | Children of prenatal helminth infected (473) *vs* uninfected (36) mothers | 0 - 36 months old, 46.4% male | *Schistosoma haematobium*, filarial, hookworm, Soil-transmitted helminths | Hib-specific IgG | M | No significant difference in IgG levels between the groups |
| *Nash et al (2017)*[24] | Randomised placebo controlled Trial | Mother child pairs from Entebbe, Uganda | Children of Albendazole+Praziquantel (348), Praziquantel only (336), albendazole only (346) *vs* placebo (349) mothers. 68% helminth infection at baseline | One year olds, gender not reported | *Mansonella perstans*, Hookworm, *Trichuris trichuria, Schistosoma mansoni, Ascaris lumbricoides, Strongloides stercoralis* | Hib-specific IgG | M | Higher Hib IgG responses (aGMR 1.51 (95% CI 1.11-2.01)) among children of strongyloidiasis infected compared to uninfected mothers. No significant difference in antibody responses among children of treated and untreated mothers |
| *Clark et al (2016)*[21] | Cohort | Mothers and their newborns recruited from Hospital Padre Alberto Buffoni (HPAB) in Quinindé,  Esmeraldas Province, Ecuador | Children of helminth infected(712) *vs* uninfected(927) mothers | 13 months old, 50.5% male | *Ascaris lumbricoides, Trichuris trichiura*, Hookworm, *Strongyloides stercoralis, Hymenolepis nana* | Hib-specific IgG | M | No significant difference (p 1.0) in Hib specific IgG levels between the two groups |
| *Malhotra et al (2015)*[22] | Cohort | Healthy pregnant women and their offspring born at the Msambweni District Hospital on the south coast of Kenya | Children of helminth infected *vs* uninfected mothers. Total sample 450 | 6 - 36 months of age, gender not reported | Lymphatic filariasis, *Schistosoma haematobium*, Hookworm, *Trichuris trichiura, Ascaris lumbricoides, Strongyloides stercoralis* | Hib-specific IgG | W | Lower IgG responses among children born to LF and hookworm infected mothers (LF: p 0.007 and p 0.03, hookworm: p 0.034 and p 0.019, at 12 and 18 months respectively). No effect of maternal schistosomiasis on IgG. |
| *McKibben et al (2011)*[26] | Cohort | Mother child pairs from coast province, Kenya | Children of helminth infected and treated (110), infected and untreated (32) vs uninfected (144) mothers | Age and gender not reported | Filariasis and intestinal helminths | Hib-specific IgG | W | Lower titers (p 0.04) at 12 months among children of infected and untreated mothers compared to uninfected and infected treated groups. No significant difference observed at 6 months. |
| **Hepatitis B : Direct helminth exposure** | | | | | | | | |
| *Riner et al (2016)*[12] | Cohort | University Students potentially from diverse geographical regions of Kenya | Helminth infected (29) *vs* uninfected adults (60) | 18 - 57 year olds, 47% male | *Schistosoma*  *mansoni* | Antibody (IgG) and cytokine (IFN- γ and IL-10 ) responses to Hep B | W | Lower anti-HBs IgG levels (p 0.038) among Sm+ group compared to the controls two weeks after the second dose of Hepatitis B. No significant difference was observed between the groups two months after the third dose. No significant differences observed for IFN- γ and IL-10. |
| *Bassily et al (1992)*[27] | Cohort | School children from Difra, Nile Delta, Egypt | *Schistosoma mansoni* infected (245) vs uninfected (152) | 6 - 12 year olds, 60.4% male | *Schistosoma mansoni* | Antibody to hepatitis B surface antigen (anti-HBs) | M | No significant difference in anti-HBs titers between infected and uninfected children |
| *Ghaffar et al (1990)*[28] | Cohort | School children from Kafr Batanone in the Nile Delta, Egypt | *Schistosoma mansoni* infected (41) vs uninfected (39) | 8 - 12 year olds, all male | *Schistosoma mansoni* | Antibody titers | M | Lower antibody titers in the infected group (67.7±74.4 mIU/ml) compared to controls (334.8±192.9 mIU/ml) 9 months post vaccination |
| **Hepatitis B : Prenatal helminth exposure** | | | | | | | | |
| *Flügge at al (2020)*[19] | Cohort | Mother child pairs from Lambaréné, Gabon | Children of Helminth infected (53) *vs* uninfected (71) mothers | Neonates, 50.8% male | *Schistosoma haematobium, Loa loa, Trichuris trichiura,* hookworm, *Ascaris lumbricoides* | Hep B-specific IgG | W | No significant difference in IgG levels between children of helminth infected and uninfected mothers at both 9 and 12 months old |
| *Nash et al (2017)*[24] | Randomised placebo controlled Trial | Mother child pairs from Entebbe, Uganda | Children of Albendazole+Praziquantel (348), PZQ only (336), albendazole only (346) *vs* placebo (349) mothers. 68% helminth infection at baseline | One year olds, gender not reported | *Mansonella perstans*, Hookworm, *Trichuris trichuria, Schistosoma mansoni, Ascaris lumbricoides, Strongloides stercoralis* | Hep B-specific IgG | M | Higher Hep B IgG responses (aGMR 1.47 (95% CI 1.11-  1.94)) among children of strongyloidiasis infected compared to uninfected mothers. No significant difference in antibody responses among children of treated and untreated mothers |
| *Malhotra et al (2015)*[22] | Cohort | Healthy pregnant women and their offspring born at the Msambweni District Hospital on the south coast of Kenya | Children of helminth infected *vs* uninfected mothers. Total sample 450 | 6 - 36 months old, gender not reported | Lymphatic filariasis, *Schistosoma haematobium*, Hookworm, *Trichuris trichiura, Ascaris lumbricoides, Strongyloides stercoralis* | Hep B-specific IgG | W | No significant differences in antibody responses to Hep B between the groups |
| *Bassily et al (1997)*[29] | Cohort | Women presenting with their two-month-old infants for immunizations in Beheira Governorate, Egypt | Infants of helminth infected (191) *vs* uninfected (194) mothers | 9 month olds, 51.4% male | *Schistosoma mansoni* | Antibody to hepatitis B surface antigen (anti-HBs) | M | No significant difference (p 0.1) in anti-HBs titers between infants of *S. mansoni* infected and uninfected mothers |
| **Pertussis : Prenatal helminth exposure** | | | | | | | | |
| *Nash et al (2017)*[24] | Randomised placebo controlled trial | Mother child pairs from Entebbe, Uganda | Children of Albendazole+Praziquantel (348), PZQ only (336), albendazole only (346) *vs* placebo (349) mothers. 68% helminth infection at baseline | One year olds, gender not reported | *Mansonella perstans*, Hookworm, *Trichuris trichuria, Schistosoma mansoni, Ascaris lumbricoides, Strongloides stercoralis* | Pertussis-specific IgG | M | Higher responses (aGMR 1.41 (95% CI 1.06-1.88)) among children of strongyloidiasis infected compared to uninfected mothers. No significant difference in antibody responses among children of treated and untreated mothers |
| *Clark et al (2016)*[21] | Cohort | Mothers and their newborns recruited from Hospital Padre Alberto Buffoni (HPAB) in Quinindé,  Esmeraldas Province, Ecuador | Children of helminth infected(712) *vs* uninfected(927) mothers | 13 months old, 50.5% male | *Ascaris lumbricoides, Trichuris trichiura,* Hookworm, *Strongyloides stercoralis, Hymenolepis nana* | Pertussis-specific IgG | M | No significant difference (p 1.0 ) in pertussis specific IgG levels between the two groups |
| **Measles : Direct helminth exposure** | | | | | | | | |
| *Tweyongyere et al (2019)*[30] | Observational cohort study combined with a randomised trial | Children from fishing communities on the Entebbe peninsula of Lake Victoria, Uganda | Helminth infected (57) vs uninfected (54) children | 3 - 5 year olds, 47% male | *Schistosoma mansoni* | Measles-specific IgG | M | Lower anti-measles IgG levels (p 0.001) among *Schistosoma mansoni* infected compared to uninfected children at 1 week post catch-up immunisation. No significant difference observed at 24 weeks post immunisation. Higher IgG (p 0.047) among *Schistosoma mansoni* infected but PZQ treated before immunisation compared to untreated. |
| **Measles : Prenatal helminth exposure** | | | | | | | | |
| *Flügge at al (2020)*[19] | Cohort | Mother child pairs from Lambaréné, Gabon | Children of Helminth infected (53) *vs* uninfected (71) mothers | Neonates, 50.8% male | *Schistosoma haematobium, Loa loa, Trichuris trichiura,* hookworm, *Ascaris lumbricoides* | Measles-specific IgG | W | No significant difference in IgG levels between children of helminth infected and uninfected mothers at both 9 and 12 months old |
| *Ondigo et al (2018)*[20] | Cohort | Pregnant women and their children from western Kenya | Children of helminth infected (42) *vs* uninfected (57) mothers | 2 year olds, gender not reported | *Schistosoma mansoni* | Measles-specific IgG | W | Lower anti-Measles IgG (p 0.023) among children of infected compared to uninfected mothers. |
| *Clark et al (2016)*[21] | Cohort | Mothers and their newborns recruited from Hospital Padre Alberto Buffoni (HPAB) in Quinindé,  Esmeraldas Province, Ecuador | Children of helminth infected (712) *vs* uninfected (927) mothers | 13 months old, 50.5% male | *Ascaris lumbricoides, Trichuris trichiura,* Hookworm, *Strongyloides stercoralis, Hymenolepis nana* | Measles-specific IgG | M | No significant difference (p 1.0 ) in measles specific IgG levels between the two groups |
| *Kizito et al*^‡^ *(2013)*[31] | Observational analysis within a RCT | Mother child pairs from Entebbe, Uganda | Children of helminth infected *vs* uninfected mothers  Total sample 711 | One year olds, gender not reported | Hookworm, *Schistosoma mansoni*, *Mansonella perstans* | Measles-specific IgG | S | No significant difference in measles specific IgG levels between infants of helminth infected and uninfected mothers |
| *Webb et al*^‡^ *(2011)*[7] | Randomised, placebo-controlled trial | Mother child pairs from Entebbe, Uganda | Children of Albendazole and Praziquantel (628), Praziquantel only (626), Albendazole only (625) *vs* placebo (628) mothers. 68% helminth infection at baseline | One year olds, gender not reported | Hookworm, *Mansonella perstans*, *Schistosoma*  *mansoni* | Measles-specific total IgG | S | No difference in Total IgG responses between infants of albendazole/praziquantel treated and untreated mothers. |
| **Polio : Direct helminths exposure** | | | | | | | | |
| *Akinwande al(2021)*[32] | Cohort | Pre-school and school children from Nigeria | *Ascaris lumbricoides* infected (23) vs uninfected (23) children | 4-15 year olds, gender not reported | *Ascaris lumbricoides* | Poliovirus-specific IFN- γ, TNF-a, IL-4, IL-10, IL-8, IL-6 and IgA | W | Higher OPV serum  IFN–γ (p<0.001) , IL-4 (p<0.001) and IL-8 (p<00.01) levels in *Ascaris lumbricoides* -infected children compared to helminth negative children after vaccination. No significant differences in TNF-a, IL-10, IL-6 and IgA |
| **Polio : Prenatal helminth exposure** | | | | | | | | |
| *Flügge et al (2020)*[19] | Cohort | Mother child pairs from Lambaréné, Gabon | Children of Helminth infected (53) *vs* uninfected (71) mothers | Neonates, 50.8% male | *Schistosoma haematobium, Loa loa, Trichuris trichiura,* hookworm, *Ascaris lumbricoides* | Polio-specific IgG | W | No significant difference in IgG levels between children of helminth infected and uninfected mothers at both 9 and 12 months old |
| *Clark et al (2016)*[21] | Cohort | Mothers and their newborns recruited from Hospital Padre Alberto Buffoni (HPAB) in Quinindé,  Esmeraldas Province, Ecuador | Children of helminth infected (712) *vs* uninfected (927) mothers | 13 months old, 50.5% male | *Ascaris lumbricoides, Trichuris trichiura,* Hookworm, *Strongyloides stercoralis, Hymenolepis nana* | Polio-specific IgA | M | Higher OPV1 IgA (p 0.0108) and OPV3 IgA (p 0.0147) levels in children born of helminth infected compared to uninfected mothers |
| **Meningococcal : Direct helminth exposure** | | | | | | | | |
| *Brückner et al (2016)*[33] | Randomised placebo controlled trial | School children from Lambaréné, Gabon | Helminth treated (48) *vs* placebo individuals (48) | 6 -10 year olds, gender not reported | *Ascaris lumbricoides, Trichuris trichiura* | Antibody responses to *N. meningitides* A and C | M | No significant difference in antibodies against *N. meningitides* A (day 28: p 0.69, day 84:p 0.08), *N. meningitides* C (day 28: p 0.3, day 84: p 0.07) between treatment and placebo groups |
| **Pneumococcal : Prenatal helminth exposure** | | | | | | | | |
| *McKittrick et al (2019)*[23] | Cohort | Pregnant women and their newborn infants enrolled at the Msambweni County  Referral Hospital antenatal clinic in Msambweni, Kenya | Children of prenatal helminth infected (473) *vs* uninfected (36) mothers | 0 - 36 months old, 46.4% male | *Schistosoma haematobium*, filarial, hookworm, Soil-transmitted helminths | Antipneumococcal antibody (IgG) responses to antigens 1, 4, 5, 6B, 7F, 9V, 14, 18C, 19F and 23F | M | Lower PnPs 23F IgG levels (p 0.047) among children born to prenatal parasite (including malaria) infected compared to uninfected mothers. Largest difference noted at 18 months. Higher PnPs 19F IgG levels (p 0.007) at 6 months and (p 0.006) at three years among children born to prenatal or at delivery parasite infected mothers. No differences observed for antigens PnPs 5, 7F and 9V |
| *Singer et al (2017)*[34] | Cohort | Children from a birth cohort in Msambweni District Hospital on the south coast of Kenya | Maternal or early childhood exposure to helminth *vs* unexposed. Total sample 281 | 4 - 7 year olds, 51% male | *Schistosoma haematobium, Trichuris trichuria, Ascaris lumbricoides, Strongyloides stercoralis*, filaria (*Wuchereria bancrofti*), Hookworm | Antigen-specific antipneumococcal  IgG | W | Higher IgG levels (p <0.05) among children with previous exposure to Trichuris at any point during infancy. No significant differences were observed for prenatal exposure to maternal parasitic infections or exposure at the time of vaccination. |
| **Ty21a : Direct helminth exposure** | | | | | | | | |
| *Bhuiyan et al (2014)*[35] | Randomised controlled trial | Children from urban area of Mirpur, Dhaka, Bangladesh | Helminth treated (126) *vs* placebo children (126). All helminth infected at baseline | 2 - 5 year olds, 54% male | *Ascaris lumbricoides, Trichuris trichiura* | Ty21a -specific  IgA, IgG and IgM | W | No significant difference (p 0.20-0.36) in plasma and ALS antibody titers between anti-helminth treated and  untreated young children post vaccination |
| *Muniz-Junqueira et al (1996)*[36] | Cohort | Individuals from Brasilia, Brasil | S. mansoni infected (26) vs uninfected (9) | 13 - 50 year olds, gender not reported | *Schistosoma mansoni* | Antibody responses to Ty21a | W | Lower Salmonella antibody responses to H antigen among infected compared to uninfected (p 0.02) individuals. No significant difference between infected and uninfected individuals for Salmonella responses to O antigen |
| **Cholera : Direct helminth exposure** | | | | | | | | |
| *Brückner et al (2016)*[33] | Randomised controlled trial | School children from Lambaréné, Gabon | Helminth treated (46) *vs* placebo individuals (43) | 6 - 10 year olds, gender not reported | *Ascaris lumbricoides, Trichuris trichiura* | Cholera-specific IgG | M | No significant difference in IgG titers (day 28: p 0.47, day 84:p 0.8) between treatment and placebo groups |
| **Rubella : Prenatal helminth exposure** | | | | | | | | |
| *Clark et al (2016)*[21] | Cohort | Mothers and their newborns recruited from Hospital Padre Alberto Buffoni (HPAB) in Quinindé,  Esmeraldas Province, Ecuador | Children of helminth infected (712) *vs* uninfected (927) mothers | 13 months old, 50.5% male | *Ascaris lumbricoides*, *Trichuris trichiura*, Hookworm, *Strongyloides stercoralis*, *Hymenolepis nana* | Rubella-specific IgG | M | No significant difference (p 1.0 ) in Rubella-specific IgG levels between the two groups |
| **Rotavirus : Prenatal helminth exposure** | | | | | | | | |
| *Clark et al (2016)*[21] | Cohort | Mothers and their newborns recruited from Hospital Padre Alberto Buffoni (HPAB) in Quinindé,  Esmeraldas Province, Ecuador | Children of helminth infected (712) *vs* uninfected (927) mothers | 13 months old, 50.5% male | *Ascaris lumbricoides, Trichuris trichiura,* Hookworm, *Strongyloides stercoralis, Hymenolepis nana* | Rotavirus-specific IgA | M | Higher rotavirus-specific IgA (p 0.0147) levels in children born to helminth infected compared to uninfected mothers |

*†‡ Results from the same study, **Risk of bias assessment based on EPHPP tool S: Strong, M: Moderate and W: Weak; PPD=purified protein derivative, cCFP=crude culture filtrate proteins of *Mycobacterium tuberculosis*, aGMR=adjusted geometric mean ratio.

**Appendix 7: Characteristics and findings from animal experiments**

| **Author (Year)** | **Vaccine** | **Animal species** | **Compared groups (sample size)** | **Age, gender** | **Type of helminth** | **Outcome** | **Finding** |
| --- | --- | --- | --- | --- | --- | --- | --- |
| **BCG** | | | | | | | |
| *Feng et al (2018)*[37] | BCG | C57BL/6 | Helminth infected and non-infected | 4 - 5 weeks, Both males and females | *Heligmosomides polygyrus* | Lymph node expansion | -Lymph node expansion in response to BCG was reduced in mice with helminth infection  -fewer mycobacteria specific CD4 T cells were found in the popliteal LN of worm infected compared to uninfected mice |
| *Obieglo et al (2016)*[38] | BCG | C57BL/6, congenic CD45.1 (Ly5.1), and P25-TCRTg RAG-12/2 (20) 3  RAG-12/2 ECFP | Helminth infected (5) vs uninfected (5) | 4 - 5 weeks, Female | *Heligmosomides*  *polygyrus bakeri*, Leishmania major | Mycobacteria-triggered Th1 responses at distal sites  DTH responses | -Reduced P25-  TCRTg and IFN-ү^+^ P25-TCRTg cells (p <0.01) among H. *polygyrus* infected compared to uninfected mice following immunisation  -No significant effect on footpad swelling was observed in mice tested for PPD reactivity 4 weeks after worm infection and 2 weeks after BCG infection, compared with BCG-infected, worm-free mice  -delayed-type hypersensitivity (DTH) responses were significantly diminished in chronic worm- infected animals compared to the un infected  -Infected animals had a 2-fold bacterial load when infected with BCG as compared to un infected animals |
| *Rafi et al (2015)*[39] | BCG | Wild-type BALB/c mice | Helminth infected vs uninfected | 6 - 8 weeks, Female | *Heligmosomides polygyrus* | Treg responses, progression of Mtb infection, BCG vaccine efficacy | No effect of prior chronic enteric helminth infection on both primary and memory T reg responses, progression of Mtb infection and BCG efficacy |
| *Al-Riyami et al (2009)*[40] | BCG (PPD) | BALB/c mice | Helminth infected vs uninfected | Age and gender not reported | Filarial Nematode | Antibody and cytokine levels, and splenic proliferation | Helminth product ES-62 did not modulate the Th1 immune response induced to PPD |
| *Elias et al (2005)*[41] | BCG | BALB/c mice | Helminth infected vs uninfected | 8 - 10 weeks, female | *Schistosoma mansoni* | Cytokine (IFN-ү, IL-4, IL-5) levels, Colony forming units | *S. mansoni* infection significantly reduced (p < 0.05) PPD specific *in vitro* IFN-ү production from splenic lymphocytes. No significant difference in PPD specific IL-4, IL-5 between the groups. Higher bacterial loads in the Schistosoma infected group compared to Schistosoma free group in lungs, liver and spleen at 6, 9 and 15 weeks post *M. bovis* BCG infection |
| *Elias et al (2005)*[42] | BCG | C57/bl mice | Helminth infected (15) vs uninfected (15) | 8 - 10 weeks, female | *Schistosoma mansoni* | Cytokine production (IFN-ү, IL-4, IL-5), nitric oxide induction | Higher (p <0.05) number of colony forming units of TB bacilli; significant reduction in nitrite levels; significant reduction in IFN-ү to PPD (p 0.05) among infected mice compared uninfected  Controls.  No significant difference in IL-4 and IL-5 to PPD between the groups |
| *Erb et al (2002)*[43] | BCG | C57BL/6 mice | Helminth infected vs uninfected | Age and gender not reported | *Nippostrongylus brasiliensis* | 1. number of M. bovis BCG bacteria present  in the lungs. 2. the antimycobacterial  Th1 response induced in the lungs. 3. Th2 (IL-4, IL-5, IL-10) responses in sups of in vitro stimulated MLN cells | Helminth infection reduced Th1 immune responses to mycobacteria in the lungs. Mycobacterial clearance was not delayed in the  Helminth infected animals compared to uninfected |
| *Pearlman et al (1993)*[44] | BCG (PPD) | BALB/c mice | Helminth infected vs uninfected | Age not reported, Female | *Brugia malayi* | Cytokine (IFN-ү, IL-4, IL-5) responses to PPD | -No difference in IFN-ү production between infected and uninfected mice following BCG. IL-4 and IL-5 production was observed in mice infected before immunisation compared to uninfected or infected simultaneously with immunisation.  -In mice immunised with BmA first and then PPD, there was a significant IL-5 production (P˂0.05) in response to PPD.  There was no PPD-specific IL-4 detected and the level of IFN-y production was similar to that from animals immunized with PPD alone |
| *Blackwood et al (1978)*[45] | BCG | ICR mice | Helminth infected vs uninfected | 8 -10 weeks, Female | *Trichinella spiralis* | Delayed-type hypersensitivity (DTH) | Delayed Hypersensitive footpad swelling (p <0.02) among infected mice vaccinated 0 or 3 days after infection compared to uninfected vaccinated mice |
| *Molinari et al (1974)*[46] | BCG | ICR mice (CD-I strain) | Helminth infected vs uninfected | 8 -10 weeks, Female | *Trichinella spiralis* | Delayed hypersensitivity after BCG immunisation (footpad swelling) | Greater footpad swelling among infected compared to uninfected controls (p 0.01) |
| *Olds et al (1969)*[47] | BCG | BALB/c strain (30 mice) and FF strain (40 mice) | Helminth infected vs uninfected | 8 weeks, Males and Females | *Hymenolepis nana* | Survival times of mice | Evidence of H. nana influence on experimental BCG. The infection caused male mice to die earlier than females after challenge with tubercle bacilli |
| **Tetanus Toxoid** | | | | | | | |
| *Kolbaum et al (2012)*[48] | 1. ACT-CSP  toxoid construct  2. live bacterial oral vaccine with  recombinant S. typhimurium SB824/pST-TB | BALB/c (H-2Kd) mice, cotton rats (Sigmodon hispidus), Wistar rats (Rattus  norvegicus) | Helminth infected vs uninfected  Total sample: 4 | Mice: 8 - 10 weeks, female  Rats: 4 – 8 weeks | *Strongyloides*  *ratti, Litomosoides sigmodontis* | P. berghei burden, T-cell production of cytokines (IFN-gamma, TNF-alpha), CSP-specific cell lysis in vivo | Higher number of IFN- γ –producing Splenocytes and percentages of CSP-specific CD81  IFN-ү/TNF-a-producing splenocytes (p <0.05) among uninfected compared to the L. sigmodontis infected group |
| *Brito et al (1976)*[49] | Tetanus Toxoid | Swiss TO mice | Helminth infected vs uninfected. Total sample: 53 | Age not reported, Male | *Schistosoma mansoni* | Antitoxin titres | Lower antitoxin titres (p <0.02) in the infected compared to the uninfected group when vaccination was done at least 9 weeks after infection. No difference in antitoxin titres between the groups when vaccination was done in the prepatent period (1-6 weeks) after infection |
| **Diphtheria** | | | | | | | |
| *Haseeb et al (1997)*[50] | Diphtheria toxoid | CF-1 mice and an albino M-line of Biomphalaria gluhrata | Helminth infected vs uninfected. Total sample: 210 | Age unclear, female | *Schistosoma mansoni* | Diphtheria toxin-neutralizing antibody | Lower antitoxin levels (p <0.05) among mice infected 16, 12, 8 and 2 weeks prior to immunisation compared to uninfected controls |
| **Influenza** | | | | | | | |
| *Stetter et al (2021)*[51] | Influenza | C57BL/6 mice | Helminth infected vs uninfected and helminth treated vs untreated | 8 - 12 weeks, gender not reported | *Litomosoides sigmodontis* | Antibody titres | Lower HI titers (<0.0001) in infected compared to uninfected mice 3 weeks after vaccination. Helminth treatment before and at the time of vaccine administration did not restore antibody response in helminth-infected mice |
| *Hartmann et al (2019)*[52] | Influenza | C57BL/6 and BALB/c Mice | Helminth infected vs uninfected | 8 - 12 weeks, both males and females | *Litomosoides sigmodontis* | Antibody (IgG1, IgG2b, IgG2c) responses to HA, HI titers | Lower HA specific antibodies and HI titers (p <0.05) in the infected compared to uninfected group |
| **Hepatitis B** | | | | | | | |
| *Guan et al (2013)*[53] | HBV | BALB/c mice | Helminth infected vs uninfected | 6 - 8 weeks, Male | *Trichinella spiralis* | Anti-HBsAg antibodies and cytokine ( IFN-ү, IL-2, IL-4, IL-10 and IL-5) levels | Higher IL-4, IL-5 (p <0.01) levels; Lower anti-HBsAg antibodies, IFN-ү and IL-2 (p < 0.01) in the enteric stage infected compared to uninfected groups. No difference in responses between muscle stage infected and uninfected groups. |
| *Chen et al (2012)*[54] | Hepatitis B | BALB/c mice | Helminth infected vs uninfected Total sample: 70 | 6 - 8 weeks, Male | *Schistosoma japonicum* | Antibody and cytokine (IFN-ү, IL-2, IL-4, IL-5, IL-10) responses to HBV | Decreased anti-HBsAg antibody, IFN-ү, IL-2 mRNA levels (p<0.05); Increased IL-4 and IL-5 mRNA levels (p <0.05) among chronically infected mice compared to uninfected controls. No significant difference between Acutely infected and uninfected controls. -After treatment with PZQ at 12 and 16 weeks, the anti HBsAg antibody of infected mice reached the control group (p>0.05) |
| **Pertussis** | | | | | | | |
| *O’neill et al (2001)*[55] | Pertussis whole cell vaccine | BALB/c mice and C57BL/6 | Helminth infected vs uninfected | 8 - 10 weeks, Female | *Fasciola hepatica* | B. pertussis specific  IFN-g, IL-4, IL-5, IL-2 | Reduced IFN-ү (p 0.01) among infected compared to uninfected mice post immunisation. No significant differences observed for IL-4, IL-5 and IL-2 |
| **Pneumococcal** | | | | | | | |
| *Apiwattanakul et al (2014)*[56] | Pneumococcal vaccine  Polysaccharide vaccines | BALB/c | Helminth infected vs uninfected | 8 weeks, Female | *Taenia crassiceps* | Antibodies (IgM, IgG1, IgG2a, IgG3) pnuemococcal colony forming uinits for Opsonophagocytic killing | Lower IgM, IgG1 and IgG3 (p < 0.05) among infected compared to uninfected mice 42 days after vaccination. Antibodies taken from Taenia-infected, vaccinated mice were unable to effectively opsonize S. pneumoniae for killing by alveolar macrophages |
| **HPV** | | | | | | | |
| *Gent et al (2019)*[57] | HPV | Olive baboon | Helminth infected (3), infected and treated with PZQ (4) vs uninfected (3). A total of 10 baboons | Sub adult, both males and females | *Schistosoma mansoni* | HPV serum specific IgG antibodies  Cytokine responses IFN-ү, IL-4 | Lower HPV specific IgG antibodies among infected compared to infected and treated (p 0.0066) and uninfected (p 0.0015) groups. No significant difference in IFN-ү and IL-4 between the groups |
| **Yellow fever** | | | | | | | |
| *Reese et al (2016)*[58] | Yellow fever virus (YFV-17D) | C57Bl/6 mice | Helminth infected vs uninfected | Age and gender not reported | *Heligomosomoides polygyrus* | IgG responses to YF, gene expression by microarray | Reduced anti-Yellow Fever IgG level (p <0.01) among chronic virus and helminth infected mice compared to uninfected controls 34-40 days after vaccination. Observed differential gene expression profiles between co-infected mice and mock-infected mice after vaccination at days 3 (rho = 0.029), 7 (rho = 0.11), and 21 (rho = 0.06). By K-means clustering five temporally distinct groups of genes were differentially expressed between mock and co-infected mice, containing a total of 701 genes that fall into a broad range of biological functions. |
| **Cholera** | | | | | | | |
| *Ljungström et al (1980)*[59] | Cholera toxin | C57BL mice | Helminth infected vs uninfected | Age and gender not reported | *Trichinella spiralis* | Intestinal secretion, absorption, in vitro IgA and IgG levels | Intestinal fluid accumulation was increased in infected compared to the control mice  Reduced IgA responses in the small intestine among infected (intestinal stage) mice compared to uninfected. No difference between the groups when vaccination was done before infection. No differences for IgG |
| **Rabies** |  |  |  |  |  |  |  |
| Mojzisova et al (2007)[60] | Antirabies vaccine (Rabisin, Merial, France) | dogs | Helminth infected (8) vs uninfected (6) | 4 months, both males and females | *Toxocara canis , Toxascaris leonina and Trichuris vulpis* | Antirabies antibody titers | Lower concentrations of anti-rabies antibodies in helminth infected puppies 28 days after vaccination (0.039-0.079 EU /ml) which are considered insufficient for protection compared to protective concentration levels in the range of 1.005-16.600EU/ml among helminth negative puppies. |

PPD=purified protein derivative, PZQ=Praziquantel, CSP=*P. berghei* circumsporozoite protein, MLN=mediastinal lymph node

**Appendix 8: Funnel plot of effect sizes for the effect of direct helminth exposure or anthelminthic treatment on vaccine responses**

Egger’s test for funnel plot symmetry resulted in a significant p value of 0.001 rejecting the hypothesis of no small study effects. The corresponding funnel plot for this result is presented in the left panel. As a sensitivity analysis, when one effect size that is extremely large [17] was left out of the analysis the resulting funnel plot on the right panel and corresponding Egger’s test resulted in a p value of 0.523 supporting evidence of funnel plot symmetry and no small study effects in the meta-analysis.

| **Includes all effect sizes in the meta-analysis** | **Excludes one extremely large effect size** |
| --- | --- |
| 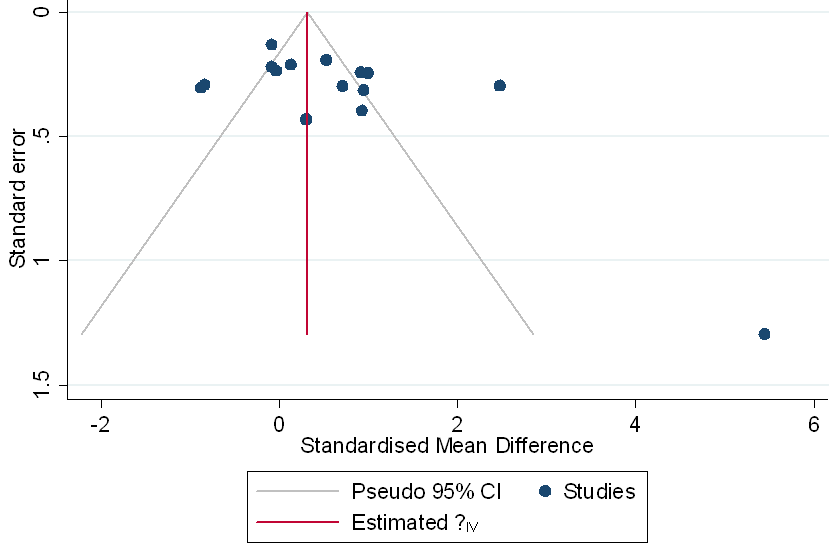 | 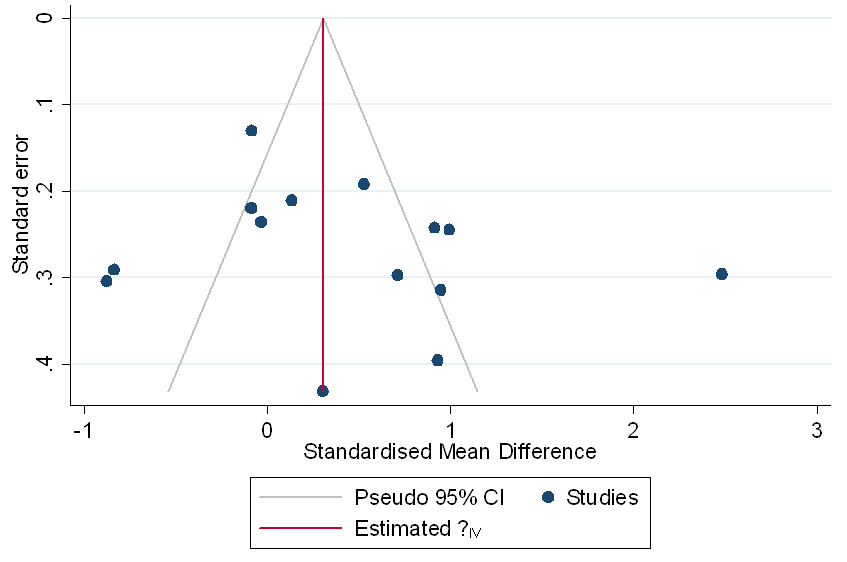 |

**Appendix 9: Funnel plot of effect sizes for the effect of prenatal helminth exposure or anthelminthic treatment on vaccine responses**

The funnel plot in the left panel includes all effect sizes included in the meta-analysis. The Egger’s test for funnel plot symmetry resulted in a significant p value <0.001 rejecting the hypothesis of no small study effects. As a sensitive analysis, one large effect size was removed [61] from the analysis and the resulting funnel plot is presented in the right panel. Egger’s test for this funnel plot symmetry resulted in a p value of 0.101.

| **Includes all effect sizes in the meta-analysis** | **Excludes one extremely large effect size** |
| --- | --- |
|  |  |

**References**

1. M Vaca A L Moncayo CACMECLRC-BDJLPJC. a single dose of oral bcg moreau fails to boost systemic ifn-γ responses to tuberculin in children in the rural tropics: evidence for a barrier to mucosal immunization. [Internet]. Vol. 2012, Journal of Tropical Medicine. 2012. p. Article-132583. Available from: http://www.hindawi.com/journals/jtm/2012/132583/

2. Elias D, Britton S, Aseffa A, Engers H, Akuffo H, D. E, et al. Poor immunogenicity of BCG in helminth infected population is associated with increased in vitro TGF-beta production. Vaccine [Internet]. 2008 Jul 23 [cited 2019 Feb 12];26(31):3897–902. Available from: http://www.ncbi.nlm.nih.gov/pubmed/18554755

3. Elias D, Wolday D, Akuffo H, Petros B, Bronner U, Britton S. Effect of deworming on human T cell responses to mycobacterial antigens in helminth-exposed individuals before and after bacille Calmette-Guérin (BCG) vaccination. Clin Exp Immunol. 2001;123(2):219–25.

4. Kilian HD, Nielsen G. Cell-mediated and humoral immune responses to BCG and rubella vaccinations and to recall antigens in onchocerciasis patients. Trop Med Parasitol [Internet]. 1989 Dec [cited 2019 Feb 12];40(4):445–53. Available from: http://ovidsp.ovid.com/ovidweb.cgi?T=JS&PAGE=reference&D=emed4&NEWS=N&AN=20060127

5. Badawy AA, Yahya RS, Awad SI, Al-Sawah GA, Kizilbash NA. Relationship between NRAMP1 gene polymorphism and efficacy of BCG vaccine in a helminth-infected population. Genet Mol Res [Internet]. 2013 Jan 30 [cited 2019 Feb 12];12(3):3048–56. Available from: http://www.geneticsmr.com//year2013/vol12-1/pdf/gmr2048.pdf

6. Ndibazza J, Mpairwe H, Webb EL, Mawa PA, Nampijja M, Muhangi L, et al. Impact of Anthelminthic Treatment in Pregnancy and Childhood on Immunisations, Infections and Eczema in Childhood: A Randomised Controlled Trial. PLoS One. 2012;7(12).

7. Webb EL, Mawa PA, Ndibazza J, Kizito D, Namatovu A, Kyosiimire-Lugemwa J, et al. Effect of single-dose anthelmintic treatment during pregnancy on an infant’s response to immunisation and on susceptibility to infectious diseases in infancy: a randomised, double-blind, placebo-controlled trial. Lancet (London, England) [Internet]. 2011 Jan 1 [cited 2019 Feb 11];377(9759):52–62. Available from: https://linkinghub.elsevier.com/retrieve/pii/S0140673610614572

8. Elliott AM, Mawa PA, Webb EL, Nampijja M, Lyadda N, Bukusuba J, et al. Effects of maternal and infant co-infections, and of maternal immunisation, on the infant response to BCG and tetanus immunisation. Vaccine [Internet]. 2010;29(2):247–55. Available from: http://dx.doi.org/10.1016/j.vaccine.2010.10.047

9. Djuardi Y, Sartono E, Wibowo H, Supali T, Yazdanbakhsh M. A longitudinal study of BCG vaccination in early childhood: The development of innate and adaptive immune responses. PLoS One. 2010;5(11).

10. Elliott AM, Namujju PB, Mawa PA, Quigley MA, Nampijja M, Nkurunziza PM, et al. A randomised controlled trial of the effects of albendazole in pregnancy on maternal responses to mycobacterial antigens and infant responses to Bacille Calmette-Guérin (BCG) immunisation [ISRCTN32849447]. BMC Infect Dis [Internet]. 2005 Dec 21 [cited 2019 Feb 12];5:115. Available from: http://www.ncbi.nlm.nih.gov/pubmed/16371154

11. Malhotra I, Mungai P, Wamachi A, Kioko J, Ouma JH, Kazura JW, et al. Helminth- and bacillus Calmette-Guerin-induced immunity in children sensitized in utero to filariasis and schistosomiasis. J Immunol [Internet]. 1999 Jun 1 [cited 2019 Feb 12];162(11):6843–8. Available from: http://ovidsp.ovid.com/ovidweb.cgi?T=JS&PAGE=reference&D=emed6&NEWS=N&AN=29309413

12. Riner DK, Ndombi EM, Carter JM, Omondi A, Kittur N, Kavere E, et al. Schistosoma mansoni Infection Can Jeopardize the Duration of Protective Levels of Antibody Responses to Immunizations against Hepatitis B and Tetanus Toxoid. MacDonald AS, editor. PLoS Negl Trop Dis [Internet]. 2016 Dec 7 [cited 2019 Feb 12];10(12):e0005180. Available from: https://dx.plos.org/10.1371/journal.pntd.0005180

13. Riet E van, Retra K, Adegnika AA, Jol-van der Zijde CM, Uh HW, Lell B, et al. Cellular and humoral responses to tetanus vaccination in gabonese children. Vaccine. 2008;26(29–30):3690–5.

14. Nookala S, Srinivasan S, Kaliraj P, Narayanan RB, Nutman TB. Impairment of tetanus-specific cellular and humoral responses following tetanus vaccination in human lymphatic filariasis. Infect Immun [Internet]. 2004 May [cited 2019 Feb 12];72(5):2598–604. Available from: http://www.ncbi.nlm.nih.gov/pubmed/15102768

15. Cooper PJ, Espinel I, Wieseman M, Paredes W, Espinel M, Guderian RH, et al. Human onchocerciasis and tetanus vaccination: Impact on the postvaccination antitetanus antibody response. Infect Immun [Internet]. 1999 Nov [cited 2019 Feb 12];67(11):5951–7. Available from: http://ovidsp.ovid.com/ovidweb.cgi?T=JS&PAGE=reference&D=emed6&NEWS=N&AN=29508123

16. Cooper PJ, Espinel I, Paredes W, Guderian RH, Nutman TB. Impaired tetanus-specific cellular and humoral responses following tetanus vaccination in human onchocerciasis: a possible role for interleukin-10. J Infect Dis [Internet]. 1998 Oct [cited 2019 Feb 12];178(4):1133–8. Available from: http://www.ncbi.nlm.nih.gov/pubmed/9806045

17. Sabin EA, Araujo MI, Carvalho EM, Pearce EJ. Impairment of tetanus toxoid-specific Th1-like immune responses in humans infected with Schistosoma mansoni. J Infect Dis [Internet]. 1996 Jan [cited 2019 Feb 12];173(1):269–72. Available from: http://www.ncbi.nlm.nih.gov/pubmed/8537675

18. Prost A, Schlumberger M, Fayet MT. Response to tetanus immunization in onchocerciasis patients. Ann Trop Med Parasitol. 1983;77(1):83–5.

19. Sandri TL, Bruckner S, Strunk J, Flugge J, Askani E, Manouana GP, et al. Impact of helminth infections during pregnancy on vaccine immunogenicity in gabonese infants. Vaccines [Internet]. 2020;8(3):1–17. Available from: https://www.mdpi.com/2076-393X/8/3/381/pdf

20. Ondigo BN, Muok EMO, Oguso JK, Njenga SM, Kanyi HM, Ndombi EM, et al. Impact of mothers’ schistosomiasis status during gestation on children’s IgG antibody responses to routine vaccines 2 years later and anti-schistosome and anti-malarial responses by neonates in western Kenya. Front Immunol. 2018;9(JUN):1–10.

21. Clark CE, Fay MP, Chico ME, Sandoval CA, Vaca MG, Boyd A, et al. Maternal Helminth Infection Is Associated With Higher Infant Immunoglobulin A Titers to Antigen in Orally Administered Vaccines. J Infect Dis [Internet]. 2016 Jun 15 [cited 2019 Feb 12];213(12):1996–2004. Available from: https://academic.oup.com/jid/article-lookup/doi/10.1093/infdis/jiw066

22. Malhotra I, McKibben M, Mungai P, McKibben E, Wang X, Sutherland LJ, et al. Effect of Antenatal Parasitic Infections on Anti-vaccine IgG Levels in Children: A Prospective Birth Cohort Study in Kenya. Ricaldi JN, editor. PLoS Negl Trop Dis [Internet]. 2015 Jan 15 [cited 2019 Mar 7];9(1):1–18. Available from: https://dx.plos.org/10.1371/journal.pntd.0003466

23. McKittrick ND, Malhotra IJ, Vu DM, Boothroyd DB, Lee J, Krystosik AR, et al. Parasitic infections during pregnancy need not affect infant antibody responses to early vaccination against streptococcus pneumoniae, diphtheria, or haemophilus influenzae type B. PLoS Negl Trop Dis. 2019;13(2).

24. Nash S, Mentzer AJ, Lule SA, Kizito D, Smits G, van der Klis FRM, et al. The impact of prenatal exposure to parasitic infections and to anthelminthic treatment on antibody responses to routine immunisations given in infancy: Secondary analysis of a randomised controlled trial. Diemert DJ, editor. PLoS Negl Trop Dis [Internet]. 2017 Feb 8 [cited 2019 Feb 12];11(2):e0005213. Available from: http://www.plosntds.org/index.php

25. Bruckner S, Agnandji ST, Berberich S, Bache E, Fernandes JFJF, Schweiger B, et al. Effect of Antihelminthic Treatment on Vaccine Immunogenicity to a Seasonal Influenza Vaccine in Primary School Children in Gabon: a Randomized Placebo-Controlled Trial. Simon GL, editor. PLoS Negl Trop Dis [Internet]. 2015 Jun 8 [cited 2019 Feb 12];9(6):e0003768. Available from: http://www.plosntds.org/index.php

26. McKibben MJ, LaBeaud D, McKibben EK, Gildengorin G, Mungai P, King C, et al. Effects of antenatal maternal parasitic treatment on infant antibody response to haemophilus influenzae type B (HIB) vaccination in a mother-child cohort in Coast Province, Kenya. In: American Society of Tropical Medicine and Hygiene 60th Annual meeting. The American Journal of Tropical Medicine and Hygiene; 2011.

27. Bassily S, Strickland GT, Abdelwahab MF, Esmat GE, Narooz S, Elmasry NA, et al. Efficacy of hepatitis-b vaccination in primary-school children from a village endemic for schistosoma-mansoni. J Infect Dis. 1992 Aug;166(2):265–8.

28. Ghaffar YA, Kamel M, Abdel Wahab MF, Dorgham LS, Saleh MS, El Deeb AS. Hepatitis B vaccination in children infected with Schistosoma mansoni: Correlation with ultrasonographic data. Am J Trop Med Hyg. 1990;43(5):516–9.

29. Bassily S, Kotkat A, Hyams KC, Youssef FG, El-Masry NA, Arthur R, et al. Immunogenicity of recombinant hepatitis B vaccine among infants of mothers with active schistosomiasis. Am J Trop Med Hyg [Internet]. 1997 Aug [cited 2019 Mar 7];57(2):197–9. Available from: http://www.ncbi.nlm.nih.gov/pubmed/9288816

30. Tweyongyere R, Nassanga BR, Muhwezi A, Odongo M, Lule SA, Nsubuga RN, et al. Effect of Schistosoma mansoni infection and its treatment on antibody responses to measles catch-up immunisation in pre-school children: A randomised trial. PLoS Negl Trop Dis [Internet]. 2019;13(2):1–17. Available from: http://dx.doi.org/10.1371/journal.pntd.0007157

31. Kizito D, Tweyongyere R, Namatovu A, Webb EL, Muhangi L, Lule SA, et al. Factors affecting the infant antibody response to measles immunisation in Entebbe-Uganda. BMC Public Health [Internet]. 2013 Jul 1 [cited 2019 Feb 12];13(1):619. Available from: http://bmcpublichealth.biomedcentral.com/articles/10.1186/1471-2458-13-619

32. K S Akinwande GOA. comparative analysis of poliovirus-specific iga and cytokine levels in the sera of ascaris lumbricoides-infected and helminth-negative nigerian children after oral poliovirus vaccination. [Internet]. Vol. 22, African Journal of Clinical and Experimental Microbiology. African Journal of Clinical and Experimental Microbiology; 2021. p. 170–8. Available from: https://www.ajol.info/index.php/ajcem/article/view/205577

33. Brückner S, Agnandji ST, Elias J, Berberich S, Bache E, Fernandes J, et al. A single-dose antihelminthic treatment does not influence immunogenicity of a meningococcal and a cholera vaccine in Gabonese school children. Vaccine [Internet]. 2016 [cited 2019 Feb 12];34(44):5384–90. Available from: http://www.ncbi.nlm.nih.gov/pubmed/27642131

34. Singer MN, Heath C, Muinde J, Gildengorin V, Mutuku FM, Vu D, et al. Pneumococcal vaccine response after exposure to parasites in Utero, in infancy, or mid-childhood. Pediatrics. 2017;139(4).

35. Bhuiyan TR, Choudhury FK, Khanam F, Saha A, Abu Sayeed M, Salma U, et al. Evaluation of immune responses to an oral typhoid vaccine, Ty21a, in children from 2 to 5 years of age in Bangladesh. Vaccine. 2014 Feb;32(9):1055–60.

36. Muniz-junqueira MI, Tavares-neto J, Prata A, Tosta CE. Antibody Response to Salmonella Typhi in human schistisimiasis mansoni. Rev Soc Bras Med Trop. 1996;29(5):441–5.

37. Feng X, Classon C, Tera G, Yang Y, Li L, Chan S, et al. Atrophy of skin-draining lymph nodes predisposes for impaired immune responses to secondary infection in mice with chronic intestinal nematode infection. PLOS Pathog. 2018;1–22.

38. Obieglo K, Feng X, Bollampalli VP, Dellacasa-lindberg I, Classon C, Helmby H, et al. Chronic Gastrointestinal Nematode Infection Mutes Immune Responses to Mycobacterial Infection Distal to the Gut. J Immunol. 2016;

39. Rafi W, Bhatt K, Gause WC, Salgame P. Neither Primary nor Memory Immunity to Mycobacterium tuberculosis Infection Is Compromised in Mice with Chronic Enteric Helminth Infection. Infect Immun. 2015;83(3):1217–23.

40. Al-Riyami L, Wilson EH, Watson CA, Harnett W. T-helper Type 1 Responses to the BCG Vaccine Component PPD in Mice Are Unaffected by the Filarial Nematode Immunomodulatory Molecule ES-62. J Parasitol [Internet]. 2009 Oct [cited 2019 Mar 7];95(5):1201–4. Available from: http://ovidsp.ovid.com/ovidweb.cgi?T=JS&PAGE=reference&D=emed11&NEWS=N&AN=358406989

41. Elias D, Akuffo H, Thors C. Low dose chronic Schistosoma mansoni infection increases susceptibility to Mycobacterium bovis BCG infection in mice. Clin Exp Immunol. 2005;398–404.

42. Elias D, Akuffo H, Pawlowski A, Haile M, Schön T, Britton S, et al. Schistosoma mansoni infection reduces the protective efficacy of BCG vaccination against virulent Mycobacterium tuberculosis. Vaccine [Internet]. 2005 Feb 3 [cited 2019 Feb 12];23(11):1326–34. Available from: http://ovidsp.ovid.com/ovidweb.cgi?T=JS&PAGE=reference&D=emed9&NEWS=N&AN=40127324

43. Erb KJ, Trujillo C, Fugate M, Moll H. Infection with the Helminth Nippostrongylus brasiliensis Does Not Interfere with Efficient Elimination of Mycobacterium bovis BCG from the Lungs of Mice. Clin Diagn Lab Immunol. 2002;9(3):727–30.

44. Pearlman E, Kazura JW, Hazlett FEJ, Boom WH. Modulation of murine cytokine responses to mycobacterial antigens by helminth-induced T helper 2 cell responses. J Immunol. 1993;151:4857–64.

45. Blackwood LL, Molinari JA. Effect of Trichinella spiralis infection on delayed hypersensitivity to heterologous antigens. Int Arch Allergy Appl Immunol [Internet]. 1978 [cited 2019 Feb 12];57(1):8–14. Available from: http://www.ncbi.nlm.nih.gov/pubmed/346495

46. Molinari JA, Cypess RH, Ebersole JL. Effect of Trichinella spiralis Infection on the Cell-Mediated Immune Response to BCG. Int Arch allergy. 1974;47:483–7.

47. Olds ARJ. The Effect of the Tapeworm Hymenolepis nana on Immunity to Tuberculosis in Mice. J Hyg (Lond). 1969;67(2):233–9.

48. Kolbaum J, Tartz S, Hartmann W, Helm S, Nagel A, Heussler V, et al. Nematode-induced interference with the anti- Plasmodium CD8 1 T-cell response can be overcome by optimizing antigen administration. Eur J Immunol. 2012;890–900.

49. Brito I V, Peel MM, Ree GH. Immunological response to Tetanus Toxoid during a schistosomal infection in mice. J Trop Med Hyg. 1976;79(7):161–3.

50. Haseeb MA, Craig JP. Suppression of the immune response to diphtheria toxoid in murine schistosomiasis. Vaccine [Internet]. 1997 Jan [cited 2019 Feb 12];15(1):45–50. Available from: http://ovidsp.ovid.com/ovidweb.cgi?T=JS&PAGE=reference&D=emed6&NEWS=N&AN=27060000

51. Nadine Stetter Wiebke Hartmann M-LBSS-BGGMB. a combination of deworming and prime-boost vaccination regimen restores efficacy of vaccination against influenza in helminth-infected mice. [Internet]. Vol. 12, Frontiers in immunology. 2021. p. 784141. Available from: http://ovidsp.ovid.com/ovidweb.cgi?T=JS&PAGE=reference&D=prem&NEWS=N&AN=34992602

52. Hartmann W, Brunn M, Stetter N, Gagliani N, Muscate F, Stanelle-Bertram S, et al. Helminth Infections Suppress the Efficacy of Vaccination against Seasonal Influenza. Cell Rep. 2019;2243–56.

53. Guan F, Hou X, Nie G, Xiao Y, Zhang Q, Liu W, et al. Effect of Trichinella spiralis Infection on the Immune Response to HBV Vaccine in a Mouse Model. Foodborne Pathog Dis. 2013 Oct;10(10):882–7.

54. Chronic Schistosoma japonicum Infection Reduces Immune Response to Vaccine against Hepatitis B in Mice. PLoS One [Internet]. 2012;7(12):e51512. Available from: http://www.plosone.org/article/fetchObjectAttachment.action?uri=info%3Adoi%2F10.1371%2Fjournal.pone.0051512&representation=PDF

55. Neill SMO, Mills KHG, Dalton JP. Fasciola hepatica cathepsin L cysteine proteinase suppresses Bordetella pertussis -specific interferon- g production in vivo. Parasite Immunol. 2001;(March):541–7.

56. Apiwattanakul N, Thomas PG, Iverson AR, McCullers JA, N. A, P.G. T, et al. Chronic helminth infections impair pneumococcal vaccine responses. Vaccine [Internet]. 2014 Sep 22 [cited 2019 Feb 12];32(42):5405–10. Available from: http://www.elsevier.com/locate/vaccine

57. Gent V, Waihenya R, Kamau L, Nyakundi R, Ambala P, Kariuki T, et al. An investigation into the role of chronic Schistosoma mansoni infection on Human Papillomavirus ( HPV ) vaccine induced protective responses. PLoS Negl Trop Dis [Internet]. 2019;1–20. Available from: http://dx.doi.org/10.1371/journal.pntd.0007704

58. Reese TA, Bi K, Kambal A, Masopust D, Haining WN, Virgin HW. Sequential Infection with Common Pathogens Promotes Human-like Immune Gene Expression and Altered Vaccine Response. Cell Host Microbe. 2016;713–9.

59. Ljungstrom I, Holmgren JAN, Huldt G, Lange S, Svennerholm A. Effect of Experimental Trichinosis on Intestinal Secretion and on Local Antibody Formation to Cholera Toxin. Scand J Infect Dis. 1980;79–81.

60. J Mojzisova J Suli MGVBSS. the effect of endoparasitism on the immune response to antirabies vaccination in puppies. [Internet]. Vol. 52, Acta Parasitologica. 2007. p. 176–80. Available from: http://www.springerlink.com/content/j176n1732126p622/?p=eac053a8e2e9444ca9e3baecd1bf7799&pi=11

61. Malhotra I, Mungai P, Wamachi A, Kioko J, Ouma JH, Kazura JW, et al. Helminth- and Bacillus Calmette-Guérin-Induced Immunity in Children Sensitized In Utero to Filariasis and Schistosomiasis. J Immunol. 1999;162:6843–8.
